# Pharmacogenetics of chemotherapy treatment response and -toxicities in patients with osteosarcoma: a systematic review

**DOI:** 10.1186/s12885-022-10434-5

**Published:** 2022-12-19

**Authors:** Evelien G. E. Hurkmans, Annouk C. A. M. Brand, Job A. J. Verdonschot, D. Maroeska W. M. te Loo, Marieke J. H. Coenen

**Affiliations:** 1 Department of Human Genetics, Radboud University Medical Center, Radboud Institute for Health Sciences, Nijmegen, The Netherlands; 2 Department of Clinical Genetics and Cardiovascular Research Institute Maastricht (CARIM), Maastricht University Medical Center, Maastricht, The Netherlands; 3 Department of Pediatrics, Radboud University Medical Center, Radboud Institute for Health Sciences, Nijmegen, The Netherlands; 4 Department of Clinical Chemistry, Erasmus University Medical Center, Rotterdam, The Netherlands

**Keywords:** Osteosarcoma, Pharmacogenetics, Cardiotoxicity, Treatment response, Hepatotoxicity, Nephrotoxicity, Bone marrow toxicity

## Abstract

**Background:**

Osteosarcoma is the most common bone tumor in children and adolescents. Despite multiagent chemotherapy, only 71% of patients survives and these survivors often experience long-term toxicities. The main objective of this systematic review is to provide an overview of the discovery of novel associations of germline polymorphisms with treatment response and/or chemotherapy-induced toxicities in osteosarcoma.

**Methods:**

MEDLINE and Embase were systematically searched (2010-July 2022). Genetic association studies were included if they assessed > 10 germline genetic variants in > 5 genes in relevant drug pathways or if they used a genotyping array or other large-scale genetic analysis. Quality was assessed using adjusted STrengthening the REporting of Genetic Association studies (STREGA)-guidelines. To find additional evidence for the identified associations, literature was searched to identify replication studies.

**Results:**

After screening 1999 articles, twenty articles met our inclusion criteria. These range from studies focusing on genes in relevant pharmacokinetic pathways to whole genome sequencing. Eleven articles reported on doxorubicin-induced cardiomyopathy. For seven genetic variants in *CELF4*, *GPR35*, *HAS3*, *RARG*, *SLC22A17*, *SLC22A7* and *SLC28A3*, replication studies were performed, however without consistent results. Ototoxicity was investigated in one study. Five small studies reported on mucosistis or bone marrow, nephro- and/or hepatotoxicity. Six studies included analysis for treatment efficacy. Genetic variants in *ABCC3*, *ABCC5*, *FasL*, *GLDC*, *GSTP1* were replicated in studies using heterogeneous efficacy outcomes.

**Conclusions:**

Despite that results are promising, the majority of associations were poorly reproducible due to small patient cohorts. For the future, hypothesis-generating studies in large patient cohorts will be necessary, especially for cisplatin-induced ototoxicity as these are largely lacking. In order to form large patient cohorts, national and international collaboration will be essential.

**Supplementary Information:**

The online version contains supplementary material available at 10.1186/s12885-022-10434-5.

## Background

Osteosarcoma is the most common primary bone tumor, that occurs most often in adolescents between the age of 10 and 25 [1, 2]. The incidence of osteosarcoma in people younger than 25 is four per 1 million [2]. Treatment consists of surgical removal of the tumor combined with systematic pre- and postoperative chemotherapy protocols. High-dose methotrexate, doxorubicin and cisplatin form the backbone of this chemotherapy treatment (MAP regimen; methotrexate, anthracycline (doxorubicin) and platinum (cisplatin) chemotherapy regimen), combined with ifosfamide or etoposide in some regimens [3]. The introduction of this chemotherapeutic treatment in the 1970s has drastically improved survival rates compared to surgery alone [1]. However, no major improvements in the treatment protocol have been made since then, with survival rates remaining 71% in the latest publications of the Euramos-1 trial [4].

Despite the positive effect of the MAP regimen on survival rates, patients also develop toxicities that can have a major effect on patients’ quality of life, during and after treatment. Moreover, severe toxicities can force oncologists to modify or even discontinue chemotherapy treatment, risking an unfavorable effect on tumor eradication. High-dose methotrexate can lead to bone marrow suppression, liver (hepato-) and renal (nephro-) toxicities [5]. The most treatment-limiting side effect of doxorubicin is cardiotoxicity, which can develop during treatment, but most often manifests more than a year after treatment [6]. Consequently, pediatric cancer survivors have a 5–15 times higher risk to develop congestive heart failure compared to the general population [7]. Lastly, cisplatin can cause acute renal damage and long-term renal insufficiency and above all, cisplatin-induced hearing loss (ototoxicity). This ototoxicity is often irreversible and has a significant impact on quality of life. Approximately 60% of all patients treated with cisplatin will develop some form of ototoxicity, of which the risk increases significantly with exposure and the final cumulative dose [8].

Gaining more insight in the cause and the development of toxicities could lead to more personalized therapies, without compromising on survival rates. Clinical factors, such as age, sex, anthracycline- and cisplatin dose and kidney function, are known to contribute to the risk of developing these different toxicities [7, 9]. Metastasis at diagnosis is the best validated predictor for treatment response [4, 10, 11]. However, this has not led to individualized treatment protocols so far. In addition, these clinical predictors do not explain all interpatient variation. Gaining more insight in genetic risk factors for survival and risk of toxicities (pharmacogenomics) may lead to a better understanding of biological mechanisms behind the different phenotypes and may ultimately lead to improved prediction models for these phenotypes seen in clinical practice.

The main goal of genetic association studies in the field of pharmacogenomics is to identify genetic variants which may explain interpatient variability in drug response, to improve drug efficacy and reduce the risk of drug-induced toxicities [12]. In patients with osteosarcoma, pharmacogenomics may also give new opportunities to optimize treatment response and reduce toxicities. Most studies in this field have been focusing on genes that were already known to be involved in the working mechanisms of the treatments used, as described previously by our group in 2016 [13]. So far, this has not resulted in the identification of genes that can be used in the clinical setting. Investigating the genetic background of treatment outcome in a non-hypothesis driven manner has the advantage that new unexpected genes might be uncovered. An illustrative example is the association between *ACYP2* and ototoxicity in patients with pediatric brain tumors which was identified in a genome-wide association study (GWAS) [14]. This association was replicated in other patients, e.g. osteosarcoma patients [15] and adult testicular cancer patients [16] and these were combined in meta-analyses [17, 18]. In all studies, patients with the AA genotype have an increased risk of ototoxicity, however the evidence is not yet strong enough to implement interventions based on this association in clinical practice. With regard to hypothesis- generating studies, osteosarcoma patients are especially suitable, because treatment has been consistent for a long time and is comparable around the world allowing the formation of larger homogenous cohorts in this relatively rare disease. The size of a homogenous patient cohort is an issue for many pediatric pharmacogenetic studies, because cohorts of patients with multiple diseases treated with similar medication are often combined. Discovery of novel associations in osteosarcoma cohorts reduces variance in the data and may accelerate the path towards the implementation to clinical practice.

This current review aims to systematically summarize the findings of hypothesis-generating pharmacogenetic studies that included patients with osteosarcoma. More specifically, we studied literature about the discovery of novel associations of germline polymorphisms with treatment response and/or chemotherapy-induced toxicities (bone marrow, hepato-, nephro-, oto-, and cardiotoxicity) in osteosarcoma patients, published in the last decade. In addition to that, replication studies of these discoveries were summarized to determine the strength of the evidence at this point in time.

## Methods

### Systematic search

The aim of this systematic review was to assess the currently available literature about the discovery of novel genetic variants involved in treatment response or treatment toxicity in patients with osteosarcoma. MEDLINE and Embase were systematically searched for relevant publications between 2010 and July 2022. The search strategy in both electronic databases consisted of three elements connected by AND, as shown in Figure S1. The first element described the patient group, which were patients with osteosarcoma. To include articles with patient cohorts with multiple diagnoses, a search term was added to describe pediatric patients with cancer, studying toxicities of cisplatin, doxorubicin or methotrexate. It was assessed manually if these mixed cohorts also include patients with osteosarcoma. The second element defined that articles should have investigated genetic variation or be genome-wide association studies. The third element defined all outcomes of interest. These include outcomes that described treatment efficacy, for example overall survival, event-free survival, disease progression or relapse. In addition, search terms were added to include toxicities of interest: ototoxicity, nephrotoxicity, bone marrow toxicity, cardiotoxicity and hepatotoxicity. These toxicities were studied, because patients with these side effects are most at risk for a dose reduction or discontinuation of treatment, which may have negative effects on treatment efficacy. In the final search strategy, all keywords with their synonyms, MeSH terms (Pubmed), emtree terms (Embase) were searched, as described in Table S1. Cochrane was searched manually with similar search words as the other databases. The date of the literature search was 30th of September 2020 and an updated search was performed on 19^nd^ of July 2022.

All articles were screened systematically by two independent reviewers (EH and AB or EH and MC for the search update) for eligibility of titles. Thereafter, abstracts were reviewed and before selection for full text, a secondary abstract selection was performed to filter for genetic association studies with available full text articles. During full text selection, the number of variants and genes that are genotyped for the publications were recorded to be able to filter out small candidate gene studies or replication studies. Genetic association studies were included if they assess more than 10 germline genetic variants in more than 5 genes in relevant drug pathways or if they use a genotyping array or other large-scale genetic analysis. The aim of the study should be to generate novel hypotheses as to which genes and/or genetic variants play a role in one of our outcomes of interest in patients with osteosarcoma. Exclusion criteria included: no osteosarcoma patients, replication studies, case reports, review articles, animal studies, in vitro studies, tumor DNA is studied or only a conference abstract is available. In case conflicts between reviewers occurred, a third independent reviewer evaluated documents leading to consensus (MC). The same reviewers (EH and AB) also independently collected and reported results (including study design, cohort size, genotyping methods, phenotype, associated variants, effect size with confidence intervals and p-values) of the studies that met the inclusion criteria.

### Quality assessment

Quality assessment was performed to assess quality of the articles that were found in this systematic review. The quality assessment form was adapted from van Vugt and colleagues (Table S5), who adapted a previously published tool [19, 20]. Briefly, the STrengthening the REporting of Genetic Association studies (STREGA) guidelines for reporting of genetic association studies was adjusted to be more applicable to pharmacogenetic studies [20, 21].

### Follow-up research

In order to find additional evidence for the associations that were found in the systematic search, a structured literature search was performed to identify studies that aim to replicate these associations. This included a search of the 91 genetic association studies identified during selection of the articles of the first systematic search performed on the 30th of September 2020. Thus the five papers identified during the updated search were not included [22–26]. In addition, PharmGKB and Pubmed were searched manually using the gene and variant of interest, doxorubicin and/or cardiotoxicity as search words. Furthermore, Web of Science was consulted to assess all articles that cited one of our selected articles. The articles that contained relevant information on replication of the associations found in the systematic search are included in the results.

## Results

### Literature search

**Fig. 1:**
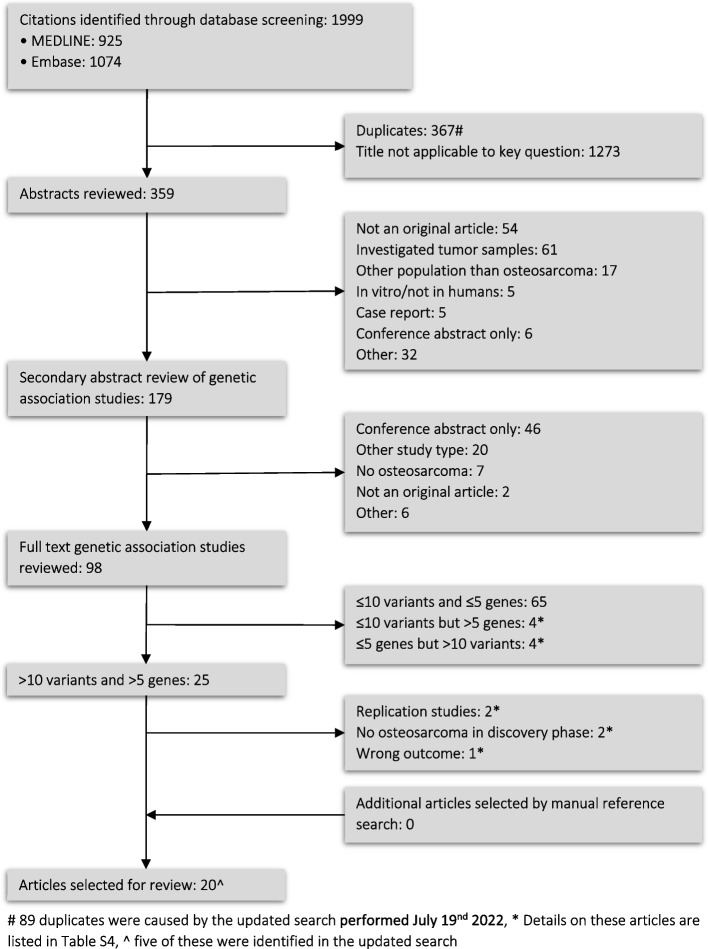
Flowchart for selection of articles

The systematic search in MEDLINE and Embase yielded a total of 1999 publications. The manual search in Cochrane yielded no additional publications. During the screening process, 367 duplicates were removed, and 1273 articles were removed because the title was not applicable to our research question (Fig. 1). Consequently, the abstract screening was performed in 2 phases. In the first phase, 180 articles were removed because they were other studies than (pharmaco) genetic association studies, and secondly, all remaining studies were assessed again to make sure the full text was available, and the study included patients with osteosarcoma. In the full-text assessment, an inventory was made of the number of variants and genes studied in each article (Table S2 and S3). Twenty-five articles, studying > 10 variants in > 5 genes, passed our inclusion criteria. Of these, two articles were excluded because they were not hypothesis-generating [27, 28], two did not have osteosarcoma patients in the discovery phase of the study [29, 30] and one article only studied methotrexate (MTX) pharmacokinetics, but not one of the outcomes of our interest [31]. Table S4 shows an overview of the articles that were excluded in the last phases of the selection as a result of the pre-defined inclusion criteria, as indicated with an asterisk in Fig. 1. Eventually, twenty articles were explored further in this systematic review.

### Quality assessment

The 20 studies that resulted from the systematic search were assessed for their quality of reporting and the results to the questions are presented in Table S6. The reporting of basic characteristics of the patient cohort was sufficient in 18 of 20 studies (90%). As shown in Table S7, these 18 studies all reported on the sex, age and diagnosis of the patients (for two studies this was only in general terms and not in a demographic table). The reporting of additional characteristics depended on the main outcome that was studied. Of the six articles on treatment efficacy, five reported on metastasis at diagnosis, four on the location of the osteosarcoma (axial or not) and four on the histological subtype of the tumor. The most important characteristics of the eight studies focusing on cardiotoxicity were follow-up time (10/11), radiation involving chest region (7/11) and anthracycline cumulative dose (8/11). Power calculations are lacking in 17 of the 20 studies (85%). Three studies performed a retrospective power calculation to explain why variants that were previously found, were not found in their study. Eleven studies contained a validation cohort to replicate their findings.

For genotyping, six studies used a genome-wide array, four studies used whole genome/exome sequencing, four studies assessed an array with variants in genes involved in absorption, distribution, metabolism and excretion (ADME) of medicines and one used a chip with genes involved in cardiovascular disease. Four studies did manual genotyping with (multiplex) allelic discrimination assays. All studies that did not analyze their data as a GWAS, had a clear rationale in their introduction or methods regarding the choice of variants (e.g. ADME or cardiovascular disease variants). Regarding quality control of the genotyping data, eleven studies reported on the exclusion of variants based on call rates, with cut-offs ranging from 0.85 to 0.99. Hardy Weinberg equilibrium (HWE) calculations were performed in 18 studies and lead to exclusion of variants in 16 of these articles if a variant deviated from HWE. During statistical analyses 16 studies assessed the effects of covariates and corrected for them in their genetic association analysis accordingly. Finally, fourteen studies used a form of correction for multiple testing, ranging from the strict Bonferroni correction to adjusting to a stricter p-value threshold without further explanation of rationale.

### Treatment response

Six of the fifteen studies identified with the systematic review focused on the role of genetic variants in treatment response or treatment efficacy of chemotherapy in patients with osteosarcoma (Table 1). A variation of treatment outcomes was used to describe treatment response, including progression free survival (PFS), event free survival (EFS), overall survival (OS), histological response, relapse and tumor necrosis.

**Table 1:** Study design, population characteristics and outcomes of hypothesis-generating pharmacogenetic studies investigating the association between genetic variants and treatment response

Author, year	Study design					Associated phenotype(s)	Associated variant(s)	Ref
	Study approach	No. of osteosarcoma patients	Follow-up time	Ethnicity; nationality	Investigated number of variants and genes			
Bhuvaneshwar et al*.,* 2019	Whole genome sequencing	INOVA: 15	NS	INOVA: NS; American	Mutation hotspot haplotypes	Relapse	Haplotypes in 26 genes	[32]
		TARGET: 85		TARGET: NS; Canadian/Brazilian			10 SNPs in 4 genes found in haplotypes in both datasets	
					4,543 variants in ADME genes	Tumor necrosis and survival	*SLC22A1* rs4646272^a^	
							*SLC22A8* rs2187384^a^	
							*UGT2B15* rs34073924^a^	
							*CHST12* rs3735099^a^	
							*CHST12* rs3735100^a^	
Caronia et al*.,* 2011	Pathway approach	102	NS	NS; Spanish	366 variants in 24 metabolism and transporter genes	OS	*ABCC3* rs4148416	[33]
						EFS	*ABCB1* rs4148737	
							*ABCB1* rs1128503	
							*ABCB1* rs10276036	
Hagleitner et al*.,* 2015	Pathway approach	Disc.: 126	NS	NS; Dutch	381 variants in 54 drug metabolism genes	PFS	*FasL* rs763110^a^	[34]
		Repl.: 64					*MSH2* rs4638843^a^	
							*ABCC5* rs939338^a^	
							*CASP3* rs2720376^a^	
							*CYP3A4* rs4646437^a^	
Hattinger et al*.,* 2016	Pathway approach	126	NS	NS; Italian	47 variants in 31 drug metabolism and transport genes	EFS	*ABCC2* rs2273697^a^	[35]
							*GGH* rs11545078^a^	
							*TP53* rs1642785^a^	
							*CYP2B6*^a^6^a^	
Koster et al*.,* 2018	GWAS	GWAS: 523	NS	GWAS: > 80% European	510,856 variants	OS	*GLDC* rs3765555	[36]
		Repl.: 109		Repl.: NS; Brazilian			*GLDC* rs55933544	
Windsor et al*.,* 2012	Pathway approach	58	41 (12–93) months	Caucasian: 41	35 variants in 21 pharmacological pathway genes of MAP	PFS	*CCND1* rs9344^a^	[37]
				Afro-Caribbean: 8			*RFC* rs1051266^a^	
				Indian/Asian: 9			*GSTT1 null*^a^	
							*GSTP1* rs1695^a^	
						Histological response	*ABCC2* rs717620^a^	
							*GSTP1* rs1695^a^	
							*MTHFD1* rs2236225^a^	

*GWAS* genome-wide association study, *INOVA* Inova pediatric group osteosarcoma patients, *TARGET* TARGET osteosarcoma dataset, *Disc.* discovery cohort, *Repl.* replication cohort, *NS* Not specified, *ADME* absorption, distribution, metabolism and excretion, *MAP* Methotrexate – Adriamycin (Doxorubicin) – Cisplatin chemotherapy regimen, *OS* overall survival, *EFS* event-free survival, *PFS* progression-free survival

^a^Association was not significant after multiple testing correction, but is/are the top hit(s) of the study

**Fig. 2:**
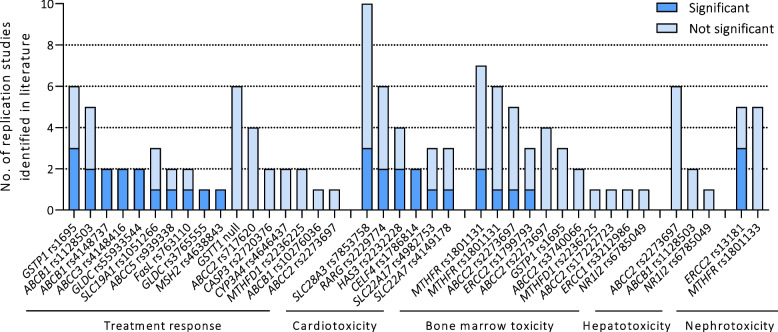
Replication studies of the association of genetic variants with treatment response and toxicities that were identified in literature. This does not include the five papers that were identified in the updated systematic search. Associations that are indicated as significant also showed consistent direction of effect with the discovery study. Table S8, S9 and S10 elaborate more on phenotypes, effect sizes and significance of these replication studies

Four of the six studies investigated treatment response in a broad panel of variants in genes involved in metabolism and transport of cisplatin, doxorubicin and methotrexate, as shown in Table 1. In the study by Caronia et al*.*, *ABCC3* rs4148416 was associated with EFS, with a hazard ratio of 6.33 (95%CI 1.79–12.7, *p* = 0.0021) [33]. This was the first evidence of the genes’ clinical relevance in osteosarcoma treatment response and this was replicated successfully in two Chinese populations with consistent directions of effects, as shown in Fig. 2 and Table S8 [38, 39]. In addition, Caronia et al. identified three variants in *ABCB1* to be associated with both EFS and OS (rs4148737, rs1128503, rs10276036) [33]. The T-allele of the *ABCB1* rs1128503 was significantly associated with improved survival. However, replication studies of this variant yielded contradictory results, namely, one study replicated this significant association with consistent directions of effect [39], two studies found significant associations in which the T-allele was a risk allele instead of a protective allele [38, 40] and two studies found no significant associations with osteosarcoma treatment response whatsoever [35, 37]. As these studies are all relatively small (Table S8) it is difficult to speculate why contradictory results occur, larger studies and meta-analysis will be necessary to shed light on this association. The second study using a pathway approach was the study by Hattinger et al*.*. They showed that *GGH* rs11545078, CYP2B6*6 and *TP53* rs1642785 and *ABCC2* rs2273697 are associated with EFS [35]. However, none of these four variants retained statistical significance in a multiparametric Cox proportional hazard regression analysis [35]. Despite that Windsor et al. could not replicate the association of *ABCC2* rs2273697 with EFS, another variant in the *ABCC2* gene, namely rs717620, was identified to be associated with poor histological response [37]. However, this association was not confirmed in four replications studies [34, 35, 38, 41]. In addition, the presence of the G-allele of *GSTP1* rs1695 was associated with poor histological response and with PFS and a variant in *RFC1/SLC19A1* (rs1051266) was significantly associated to PFS. Three of the six studies that also assessed *GSTP1* rs1695 and one of three studies that studied *RFC/SLC19A1* rs1051266 in association to EFS or OS, also found a significant association, as shown in Fig. 2 and Table S8 [35, 39–45]. Lastly, the study performed by Hagleitner et al*.* included a discovery cohort of 126 osteosarcoma patients and a replication cohort of 64 patients [34]. Five variants were identified to be significantly associated with PFS (Table 1), including *FasL* rs763110, *MSH2* rs4638843, *ABCC5* rs939338, *CASP3* rs2720376, *CYP3A4* rs4646437. Genetic risk scores were generated based on these five variants, using the number of unfavorable alleles patients had for these variants. This risk score was able to distinguish between patients with good and poor outcome, both in patients with and without metastases [34]. In the replication cohort by Xu et al., only *FasL* rs763110 and *ABCC5* rs939338 contributed to the risk score to predict treatment outcome [46]. Overall, all studies that used a pathway approach found novel genetic variants that may play a role in the response to treatment of osteosarcoma, however, the study by Caronia et al*.* was the only one large enough to correct for multiple testing.

A genome-wide association study (GWAS) including 523 osteosarcoma patients was performed by Koster et al*.* in 2018. They investigated 510,856 genetic variants in relation to OS [36]. The variant which had the strongest association with OS, namely rs1030228, could not be replicated in their replication cohort, consisting of 109 osteosarcoma patients. However, in a combined analysis of the discovery cohort and replication cohort, another variant, rs3765555, was inversely associated with OS (HR = 1.76 per copy of the A-allele). Imputation of the region centered around this variant identified a second variant, *GLDC* rs55933544, significantly associated with OS (HR = 1.92 95% CI 1.53–2.41, *p* = 1.34 × 10^–8^). Lin et al. replicated the association of rs55933544 with OS in their patient cohort with an odds ratio of 2.98 (95% CI 1.87–4.96, *p* < 0.001). Furthermore, expression quantitative trait locus (eQTL) analysis showed that the T-allele of rs55933544 was significantly associated with a decreased *IL33* expression and lower *IL33* expression was independently associated with worse osteosarcoma patient survival [36]. The study by Bhuvaneshwar et al*.* used whole genome sequencing data to identify haplotypes associated with relapse [32]. Using both the TARGET and INOVA patient datasets, 231 haplotypes were described of which the variants could be mapped to 26 genes. From the haplotypes, only four variants in *MKI67,* one in *CACNA2D4,* three in *SLC13A2* and two in *PPP1R12C* were associated with relapse in both patient datasets independently. These variants were not previously indicated in osteosarcoma treatment response and no replication studies have been performed yet. Bhuvaneshwar et al*.* also extracted variants in ADME genes from their dataset to investigate the association with tumor necrosis and OS. A total of 281 variants were associated with tumor necrosis and five of these variants, in *SLC22A1*, *SLC22A8*, *CHST12* and *UGT2B15,* were also associated with OS, prioritizing these for future research.

### Doxorubicin-induced cardiotoxicity

Eleven studies reported on pharmacogenetics concerning doxorubicin-induced cardiotoxicity (Table 2). These range from small studies with limited numbers of patient and small numbers of variants studied to GWASs and genome/exome sequencing with larger patient cohorts. In an exploratory study by Windsor et al., 36 genetic variants in 21 genes in the pharmacokinetic pathways of cisplatin, doxorubicin and methotrexate were investigated in relation to multiple outcomes in a patient cohort of 58 osteosarcoma patients [37]. With regard to doxorubicin-induced cardiotoxicity, the G-allele of *GSTP1* rs1695 was associated to both early and end-of-treatment cardiotoxicity. This variant was previously mainly indicated in treatment efficacy, as described above, however not in cardiotoxicity. Due to the exploratory nature of this study and the small patient cohort, these associations were not corrected for multiple testing. No replication studies of this association were identified in our search (Table S9). In another study, Hildebrandt et al*.* assesses 12 loci that were previously indicated in hypertension by a GWAS [47]. They found that the G-allele of *PLCE1* rs932764 and the G-allele of *ATP2B1* rs17249754 are protective to doxorubicin-induced cardiotoxicity in pediatric cancer survivors. In addition, they showed that doxorubicin exposure to iPSC-cardiomyocytes is associated to decreased *PLCE1* expression and increased *ATP2B1* expression in a dose-dependent manner.

**Table 2:** Study design, population characteristics and outcomes of hypothesis-generating pharmacogenetic studies investigating anthracycline-induced cardiotoxicity in patients with osteosarcoma

Author (year)	Study design					Associated variant(s)	Ref
	Study approach	No. of osteosarcoma patients	Follow-up time (years, or specified otherwise)	Ethnicity; nationality	Investigated number of variants and genes		
Aminkeng et al*.,* 2015	GWAS	Stage 1: 11 (of 280)	Cases: 7.5 (2.5 – 15.5) Controls: 9 (7 – 12)	Caucasian; Canadian	738,432 variants	*RARG* rs2229774	[48]
		Stage 2: 9 (of 96)	Cases: 22 (19 – 25) Controls: 17 (14 – 22)	Caucasian; Dutch			
		Stage 3: 5 (of 80)	Cases: 15.5 (7 – 22) Controls: 10 (7 – 15)	African, East Asian and Aboriginal			
Chaix et al., 2020	Exome sequencing	Disc.30^a^ of 289	Cases: 8.5 (5.0 – 12.3) Controls: 9.0 (6.0 – 12.3)	NS	110,558 variants in 17,382 genes	n/a	[24]
Hildebrandt et al*.,* 2017	Hypertension loci	41 (of 108)	Cases: 21.2 (SD: 11.2) Controls: 15.7 (SD: 7.6)	White, hispanic or black; American	12 variants in *PLCE1, ATP2B1, ARHGAP42, GNAS-EDN3, C10orf107, CSK, BAG6, CACNB2, MTHFR, CACNB2, HFE, NPR3*	*PLCE1* rs932764^*^	[47]
						*ATP2B1* rs17249754^*^	
Ruiz-Pinto et al*.,* 2017	Exome array with low frequency variants	15 (of 93)	Cases: 10.5 (1 – 27.5) Controls: 8.3 (1 – 24.1)	NS; Spanish	247,870 variants	*GPR35* rs12468485	[49]
Sapkota et al*.*, 2021	Whole genome sequencing	NS	NS	Disc.: African	9.3 million common variants (MAF ≥ 0.05) and 10.2 million rare/low frequency variants (MAF ≤ 0.05)	1p13.2 rs6689679	[23]
				Repl.: European		15q25.3 rs9788776	
Sapkota et al*.*, 2021	Whole genome sequencing	NS	NS	Disc.: European	6.69 million common variants (MAF ≥ 0.05) and rare/low frequency variants (MAF ≤ 0.05) number NS	6p21.2 rs2815063	[22]
				Repl 1.: African			
				Repl. 2.: European			
Visscher et al*.,* 2012	ADME panel	Disc.: 11 (of 156)	Cases: 6.5 (0.1 – 21.5) Controls: 7.8 (5.0 – 17.9)	Disc. and repl.: (non-) European; Canadian Repl.2: NS; Dutch	1,931 variants in 220 drug bio-transformation genes	*SLC28A3* rs7853758	[50]
		Repl.: 10 (of 188)	Cases: 7.4 (0.2 – 20.7) Controls: 9.2 (5.0 – 18.6)				
		Repl.2: 6 (of 96)	Cases: 20.2 (7.4 – 27.9) Controls: 15.4 (5.1 – 29.8)				
Visscher et al*.,* 2015	ADME panel	Discover.: 21 (of 344)	NS	NS; Canadian and Dutch	4,153 variants in 300 pharmacokinetics and -dynamics genes	*SLC22A17* rs4982753^*^	[51]
		Repl.: 16 (of 218)				*SLC22A7* rs4149178^*^	
Wang et al*.,* 2014	Cardiovascular panel: gene – environment interaction	Disc.: 54^a^ (of 287)	Cases: 10.0 (0.1 – 35.1) Controls: 11.3 (0.9 – 41.0)	Disc.: Non-Hispanic white; American	34,912 variants in 2,100 genes associated with cardiovascular disease	*HAS3* rs2232228	[52]
		Repl.: 0 (of 76)	4.0 (0.5 – 22.5)				
				Repl.: Non-Hispanic white, Hispanic, other; American			
Wang et al*.,* 2016	GWAS: gene – environment interaction	Disc.: 96^b^ (of 331)	Cases: 9.4 (0.1–35.1) Controls: 12.9 (1.4–41)	Disc.: Non-Hispanic white; American	709,358 variants	*CELF4* rs1786814	[53]
		Repl.: 17 (of 54)					
				Repl.: Non-Hispanic white, Hispanic, Other; American			
Windsor et al*.,* 2012	Pathway approach	58 (of 58)	41 (12–93) months	Caucasian, Afro-Caribbean, Indian, Asian; UK	35 variants in 21 pharmacological pathway genes of MAP	*GSTP1* rs1695^*^	[37]

*NS* not specified, *n/a* not applicable, *GWAS* genome-wide association study, *ADME* absorption, distribution, metabolism and excretion, *disc.* discovery cohort, *repl.* replication cohort, *MAP* methotrexate, adriamycin (doxorubicin), cisplatin chemotherapy regimen

^a^Number of bone tumors, osteosarcoma is not specified

^b^Number of sarcomas, osteosarcoma is not specified

^*^Association was not significant after multiple testing correction, but is/are the top hit(s) of the study

In two pharmacogenetic studies by Visscher et al*.*, both studying a broad panel of ADME genes, multiple variants in the Solute Carrier (SLC) family were significantly associated with anthracycline-induced cardiotoxicity. In their patient cohorts, approximately 80% of patients was treated with doxorubicin and the rest was treated with other anthracyclines depending on the cancer type and its treatment protocols, which was most often daunorubicin. In the first study in 2012, the A-allele of *SLC28A3* rs7853758 was found more often in controls than in cases, and significantly associated in a protective manner [50]. Another variant in the same gene (rs4877847) was also significantly associated with anthracycline-induced cardiotoxicity, even after conditioning for rs7853758 thus suggesting an independent effect. The association of the top-hit (rs7853758) was replicated in the same study in an independent patient cohort. In addition, Visscher et al*.* published a validation study in 2013 in which this effect was strengthened in a meta-analysis [27]. The additional power of this meta-analysis also gained a novel top-hit association. The A-allele of *UGT1A6* rs17863783 was found to be significantly associated with anthracycline-induced cardiotoxicity (OR (95%CI) = 4.30 (1.97–9.36), *p* = 2.4 × 10^–4^). Of the additional nine patient cohorts that attempted to replicate the *SLC28A3* rs7853758 association [54–57], only two cohorts succeeded, as shown in Fig. 2 and Table S9 [29, 49]. Interestingly, the associations were only found in pediatric patient cohorts and the effect was never found in adults. In their second discovery study, Visscher et al. studied a more broad ADME panel, containing 4153 variants in 300 genes. For both *SLC22A17* rs4982753 and *SLC22A7* rs41491178, the minor allele was found more often in controls than in cases and had a protective effect for developing cardiotoxicity after doxorubicin treatment [51]. Both variants were not statistically significantly after a strict Bonferroni correction, but the two variants together significantly improved the genotype-guided risk prediction model. In addition, the associations were successfully replicated in an independent patient cohort in the same study. However, the associations were not found in a subsequent candidate gene study [29] and GWAS [58].

A study by Wang et al*.* focused on genes that were previously associated with cardiovascular disease, since cardiotoxicity risk is influenced by coexistence of cardiovascular disease risk factors like hypertension and diabetes [52]. In this study, cumulative anthracycline exposure was calculated by multiplying the cumulative dose of individual anthracyclines with a factor that describes the drug’s cardiotoxic potential, but it was not indicated what percentage of the cohort received which anthracycline. Despite that, for this systematic review it was assumed that the majority was treated with doxorubicin. No variants were associated with anthracycline-induced cardiomyopathy, but a gene-environment interaction analysis identified the variant *HAS3* rs2232228. Among patients with the GG genotype for this variant, cardiomyopathy was infrequent and not dose related. However, the AA genotype conferred an 8.9-fold increased cardiomyopathy risk when also exposed to anthracycline doses > 250 mg/m^2^, compared to the GG genotype. In the GWAS that Wang et al. executed two years later, they found no variants associated with anthracycline-induced cardiomyopathy [53]. Again, they carried out a gene-environment analysis, which identified *CELF4* rs1786814 to have a gene-environment interaction with anthracycline dose. Patients with the *CELF4* rs1786814 GG genotype who were exposed to anthracycline levels greater than 300 mg/m^2^, had a 10.2-fold increased risk of cardiomyopathy compared to patients with the GA/AA genotype and exposure to anthracycline levels of 300 mg/m^2^ or lower. Among other variants, *HAS3* rs2232228 and *CELF4* rs1786814 were studied by Leger et al. in hematopoietic cell transplantation survivors treated with anthracyclines. For the association of *CELF4* rs1786814 with cardiomyopathy, the interaction between SNP and anthracycline dose was found, with *p* = 0.02 [59]. In addition, a significant association was found with an analysis limited to anthracycline doses > 300 mg/m^2^ (1-sided *p* = 0.01; Table S9). The interaction of *HAS3* rs2232228 AG genotype and anthracycline dose had a 1-sided *p* = 0.01 in this study. When restricting the analysis to anthracycline doses > 250 mg/m2, a significant association was found (Table S9). However, no significant association was found when studying the main effects of the variants in a complete patient cohort containing all dosages with short- or long term cardiomyopathy, for neither *HAS3* rs2232228 and *CELF4* rs1786814.

In a GWAS by Aminkeng et al*.* an association was found which was also replicated in their two replication patient cohorts [48]. In all cohorts, the majority of patients was treated with doxorubicin, followed by daunorubicin and epirubicin. The SNP, *RARG* rs2229774, was associated with anthracycline-induced cardiotoxicity for both patients receiving a low to moderate anthracycline dose and patients receiving a high anthracycline dose. Overall, rs2229774 carriers (AA or AG genotype) had significantly increased odds of developing cardiotoxicity in comparison to non-carriers after doxorubicin treatment (OR (95%CI) = 4.7 (2.7–8.3), *p* = 4.3 × 10^–11^). The only other replication study that found a significant association, was the study by Schneider and colleagues in 2017 [58]. However, they found that the A-allele causes a decreased risk rather than an increased risk for cardiotoxicity (Table S9). They argue that difference in direction of the effect may be due to the general heterogeneity between studies, for example due to drug type, drug exposure, phenotype definition and population.

The genome-wide analysis of Ruiz-Pinto et al*.* in 93 pediatric cancer survivors (15 with osteosarcoma), focused on low frequency exome variants. They used an exome array that is enriched with low frequency variants (80% of variants with minor allele frequency (MAF) ≤ 1%) [49]. No variant showed a significant association with chronic anthracycline-induced cardiotoxicity after correction for multiple testing, but a novel significant association for *GPR35* was identified by gene-based testing. The SNP rs12468485 made the greatest contribution toward the observed association. The T-allele was almost exclusively found in cases, and 67% of cases carrying the CT genotype had an extreme chronic cardiotoxicity phenotype. Up to now, no replications of this association have been attempted.

Two studies by Sapkota *er al.* whole-genome sequencing data was used [22, 23]. In the first study they specifically searched for variants associated with therapy related cardiomyopathy in childhood cancer survivors of African ancestry (*n* = 246) as in general the prevalence of non-ischemic cardiomyopathy is higher in these individuals [23]. The type of anthracyclines used was not specified. Two loci (1p13.2 and 15q25.3) showed genome-wide significant association with ejection fraction. The minor alleles resulted in a reduced ejection fraction of 4 and 5.9% respectively. One of the nine significant SNPs in the 1p13.2 locus (rs6689879) could be replicated in a cohort of patients from European ancestry although the effect was much smaller. The variant in the 15q25.3 locus could not be replicated as the variant did not exist in the European cohort. Both variants were also associated with cardiomyopathy with a 3.73 fold increased risk for grade 2–4 cardiomyopathy for the variant on chromosome 1 and a 5.24 fold increased risk for the chromosome 15 variant in cancer survivors of African ancestry. The same direction of effect was observed in patients from European ancestry for the variant on chromosome 1 but this was not statistically significant. Based on additional studies the authors suggest that the effect of rs6689879 on chromosome 1 is most likely due to a dysregulation of the *PHTF1* gene. In the second study the survivors of European ancestry (*n* = 1870) were used as discovery cohort [22]. First an association analysis including common variants from the whole-genome sequencing dataset was performed using ejection fraction as continuous outcome resulting in the identification of one genome-wide significant association (rs2815063 on chromosome 6p21.2). The analysis including rare variants did not lead to the identification of significant associations with ejection fraction. The results could be replicated in the survivors of African ancestry. The variant was also associated with cancer treatment induced cardiac dysfunction both for CTCAE grade 2 or higher and grade three and higher. The same results were found in a second cohorts of cancer survivors from European ancestry (*n* = 4020), however, the results could not be replicated in the small cohort of African ancestry. Interestingly the variant showed strongest association in survivors treated with doxorubicin only and was not found in survivors exposed to daunorubicin only. Based on methylation and gene expression analysis the authors conclude that the SNP most likely dysregulates the *KCNK17* gene. In addition, 10 previously reported SNPs associated with cancer treatment induced cardiac dysfunction were investigated. Three variants showed nominal significant association. The variant rs4149178 on *SLC22A7* was associated with increased ejection fraction in survivors exposed to anthracyclines and/or chest radiation. Analysis in survivors treated with anthracyclines only a statistically significant interaction effect on ejection fraction was found between rs2232228 in *HAS3* in survivors exposed to more than 250 mg/m^2^ anthracyclines. Finally, a nominal significant association was found for rs2229774 in *RARG* and cancer treatment induced cardiac dysfunction.

A different approach to identify genes associated with cardiotoxicity in cancer survivors was applied by Chaix et al. [24]. A variant burden analysis was performed by collapsing all rare and low frequency variants in a gene as detected by exome sequencing. This analysis led to the identification of 31 genes associated with cardiotoxicity, however none of these genes reached exome-wide significance. Overall the variant burden was lower for the cases compared to controls suggesting that the variants protect against cardiotoxicity. Most of the prioritized genes were involved in the PI3K/AKT/mTOR and p53 pathways. Six pathways were identified that were differentially enriched between cases and controls, functional follow-up of some of the genes showed that *PI3KR2* and *ZNFB827* are involved in autophagy, a relevant pathway involved in anthracycline cardiotoxicity*.* In addition, an exploratory risk prediction model was developed including clinical and genetic factors, this model showed a better risk prediction compared to the model including only clinical factors.

### Cisplatin-induced ototoxicity

In this systematic review, one publication was identified that studied the association of genetic variants with cisplatin-induced ototoxicity in patients with osteosarcoma (Table 3). Meijer et al. performed a GWAS in a cohort of 390 childhood cancer patients [25]. About 50% of the patients had osteosarcoma and ototoxicity was scored using the Muenster classification. In the discovery cohort eight suggestively significant loci were identified. Replication in two additional cohorts showed evidence for association for the variant rs893507 in the *transcription elongation regulator 1 like* (*TCERG1L*) gene. Patients carrying the C allele have an odds of 3.11 to develop cisplatin induced hearing loss. This study included functional studies to the identified gene to ototoxicity. Besides, windsor et al. planned on including ototoxicity in the genetic association study described above [37]. However, this analysis was not performed because of incomplete data.

**Table 3:** Study design, population characteristics and outcomes of hypothesis-generating pharmacogenetic studies investigating bone marrow- hepato- oto- nephrotoxicity or mucositis in patients with osteosarcoma

Author, year	Study design						Associated phenotype(s)	Associated variant(s)	Ref
	Study approach	No. of osteosarcoma patients	Follow-up time	Ethnicity; nationality	Investigated number of variants and genes	Investigated phenotypes			
Gong et al., 2021	Pathway approach	7 of 80	NS	NS; Chinese	23 variants in 15 drug metabolizing and transport genes	Mucositis	Mucositis	*ABCB1* rs1128503	[26]
								*ABCB1* rs1045642	
								*MTHFR* rs1801133	
Hattinger et al., 2016	Pathway approach	57	NS	NS; Italian	45 variants in 31 drug metabolism and transport genes	Leukopenia, thrombocytopenia, red blood cell transfusion, platelet transfusion, hepatotoxicity	Leukopenia	*ABCC2* rs2273697^a^	[35]
								*MTHFR* rs1801131^a^	
							Thrombocytopenia	*ABCC2* rs2273697^a^	
								*XPD* rs1799793^a^	
							Hepatotoxicity	*ABCB1* rs1128503^a^	
								*ABCC2* rs2273697^a^	
								*GGH* rs1800909^HWa^	
Hegyi et al., 2017	Pathway approach	59	NS	NS; Hungarian	29 variants in *ABCB1, ABCC1, ABCC2, ABCC3, ABCC10, ABCG2, GGH, SLC19A1, NR1I2*	Leukocyte/ neutrophil granulocyte count	Myelotoxicity	*ABCC2* rs2273697^a^	[60]
								*ABCC2* rs3740066^a^	
								*NR1I2* rs3732361^a^	
								*NR1I2* rs3814058^a^	
								*NR1I2* rs6785049^a^	
							Hepatotoxicity	*NR1I2* rs3732361^a^	
								*NR1I2* rs3814058^a^	
								*NR1I2* rs6785049^a^	
Hurkmans et al., 2020	ADME panel	113	NS	Caucasian; Dutch, Spanish, Australian	1936 variants in 231 ADME genes	Creatinine, ALAT, ASAT, hemoglobin, thrombocyte, leukocyte and neutrophil counts	Thrombocyte counts	*CYP2B6* rs4803418	[61]
								*CYP4F8* rs4808326	
								*CYP2B6* rs4803419	
Meijer et al., 2021	GWAS	Stage 1: 208 (of 390)	Cases: 0.4 (0–3) years Controls: 0.3 (0–2.5)	NS; European	NS	Ototoxicity	Ototoxicity	*TCERGL1* rs893507^a^	[25]
		Stage 2: 49 (of 192)	Cases: 0.7 (0.2–11.4) Controls: 0.8 (0.1–16.2)	NS; Canadian					
		Stage 3: 111 (of 188)	Cases: 1.6 (0–17.2) Controls 1.7 (0–11.8)	NS; European					
Windsor et al., 2012	Pathway approach	58	41 (12–93) months	Caucasian: 41	36 variants in 21 pharmacological pathway genes of MAP	Anemia, leucopenia, myelo-suppression, GFR, infection	Leucopenia	*ERCC1* rs3212986^a^	[37]
				Afro-Caribbean: 8				*GSTP1* rs1695^a^	
				Indian/Asian: 9				*ABCC2* rs17222723^a^	
							Anemia	*MTHFD1* rs2236225^a^	
								*MTHFR* rs1801131^a^	
								*CYBA* rs4673^a^	
							Infection	*XPC* rs2228001^a^	
							Nephrotoxicity	*ERCC2* rs13181^a^	
								*MTHFR* rs1801133^a^	

*NS* Not specified, *ADME* absorption, distribution, metabolism and excretion, *MAP* Methotrexate – Adriamycin (Doxorubicin) – Cisplatin chemotherapy regimen, *ALAT* alanine aminotransferase, *ASAT* aspartate aminotransferase

^a^Association was not significant after multiple testing correction, but is/are the top hit(s) of the study

### Bone marrow- hepato- nephrotoxicity and mucositis

Four studies were identified that focused on nephrotoxicity, hepatotoxicity and/or bone marrow toxicity in the systematic search. Table 3 shows that studies by Hattinger et al., Hegyi et al., Windsor et al. are among the smallest studies in this review, as they studied 45, 29 and 36 variants in cohorts of 57, 59 and 58 osteosarcoma patients, respectively [35, 37, 60]. Whereas significant associations were found, none of these studies corrected for multiple testing and that was also reflected by many replication studies with negative results. The results of the *ABCC2* gene is a clarifying example for this. rs2273697 was associated with hepatotoxicity and thrombocytopenia by Hattinger et al., however, as Fig. 2 indicates, six studies that also related this variant to hepatotoxicity did not find a significant association [35, 37, 41, 60–63], negative results were also found in four studies that related this variant to thrombocytopenia [35, 37, 61–63]. In addition, rs2273697 was found to be associated with leukopenia according to both Hattinger et al. and Hegyi et al., but this association was not found in 4 other cohorts [35, 37, 60–63]. Lastly, Hegyi et al. and Windsor et al. found that *ABCC2* variants rs3740066 and rs17222723, respectively, were associated with leukopenia. However these associations were not replicated in any of the replication studies [35, 37, 60, 61]. Altogether, multiple replication studies were performed of the initial findings, but none of the replication studies could confirm these (Table S10).

The association of *MTHFR* variant rs1801131 with anemia that was identified by Windsor et al., was confirmed in two cohorts of acute lymphatic leukemia patients, but the association was not significant in five other studies [37, 64–70]. In addition, Hattinger et al. showed that this variant is also associated to leukopenia. Whereas ten studies also assessed this association, there was only one study with a significant result, however with an opposite direction of effect [35, 64–66, 69–75]. The only association that shows consistent replication is the association of *ERCC2* variant rs13181 with nephrotoxicity, that was identified by Windsor et al.. In four of six patient cohorts treated with cisplatin-based treatment, the association of the AC or CC genotype was associated with increased risk to develop nephrotoxicity was confirmed, with odds ratios ranging from 3.16 to 4.4 [37, 76–80].

Finally, the study by Hurkmans et al. assessed a panel of 1936 variants in 231 genes involved in absorption, distribution, metabolism and excretion of medicines for multiple toxicities [61]. Three variants in the Cytochrome P450 family were significantly associated with thrombocyte count, namely *CYP4F8* rs4808326, *CYP2B6* rs4803418 and *CYP2B6* rs4803419, and these remained significant after Bonferroni correction for multiple testing. The two variants in *CYP2B6* were in high linkage disequilibrium, and thus likely represent the same locus. Regarding *CYP4F8* rs4808326, carriers of the A-allele had higher thrombocyte counts after methotrexate infusion compared to carriers of the G-allele. The gene has not been linked to methotrexate or thrombocyte count before. The underlying mechanisms of the associations with all three variants are still unclear [61]. These associations are not replicated in other patient cohorts yet, as the publication was recent.

One study was identified that focused on MTX induced mucositis [26]. The authors investigated 80 patients for 23 variants in 15 genes known to be involved in MTX processing in the body. Two variants in ABCB1 (rs1128503 and rs1045642) and one variant in MTHFR (rs1801133) showed association with MTX-induced mucositis in this Chinese cohort. Also this cohort is small and no correction for multiple testing was performed.

## Discussion

This systematic review provides an overview of hypothesis-generating pharmacogenetic studies in osteosarcoma patients of the last 10 years. In addition, replication studies of top-hit associations of the studies were identified in a structured manner to give a more complete idea of the evidence that is present. Treatment response and doxorubicin-induced cardiotoxicity are the most extensively studied phenotypes. Chemotherapy-induced nephrotoxicity, hepatotoxicity and bone marrow toxicity were examined, but only in small patient cohorts. The influence of genetic variants on cisplatin-induced ototoxicity was only investigated in one hypothesis-generating study.

The only GWAS that was performed in relation to treatment response to chemotherapeutic treatment in osteosarcoma patients was the GWAS by Koster et al. [36]. In that study, two variants in in the *GLDC* gene were found to be associated to overall survival. As previously mentioned, Lin et al. successfully replicated the association of rs55933544 with decreased overall survival in their patient cohort [81]. Noteworthy, the TT genotype of this variant was not related to *GLDC* expression, but it was associated to lower expression of *interleukin-33* (*IL33*) [36, 81]. On top of that, Kang and colleagues genotyped common variants in the *IL33* gene and found that the A-allele of rs1048274 was associated to survival in a osteosarcoma patient cohort of Chinese ancestry [82]. The patient cohort of Koster et al. consisted of European and Brazilian subjects and they did not identify statistically significant associations with common variants in *IL33*. Differences in linkage disequilibrium structures between populations allowed for different variants on the same locus to be associated to survival of patients with osteosarcoma, indicating that not *GLDC*, but *IL33* is causal for decreased survival through the variant. This emphasizes that studying populations of different ethnicities helps in fine mapping the genetic background that causes a phenotype. *IL33* was previously associated to prognosis in other cancers [83–85] and it is known to have pro- and anti-tumorigenic properties mediated through immune cells [86]. In osteosarcoma, *IL33* plays a role in osteosarcoma cell viability in in vitro experiments mediated through the PI3K/AKT pathway [87, 88]. However, the exact role and the effects of genetic variants remains to be found.

A pathway approach including genes linked to osteosarcoma treatment was used by most of the included studies, so consequently members of the *ABC* transporter family were included in the pharmacogenetic investigations and sometimes found to be associated to the outcomes of interest. These genes code for membrane-bound proteins which participate in the movement of most drugs and their metabolites across cell surface and cellular organelle membranes. Defects in these genes can be important in terms of cancer therapy and pharmacokinetics [89]. As indicated in the results of this review, *ABCC2* variants were repeatedly associated to toxicities in these studies, however Fig. 2 shows that these associations were scarcely replicated. Doxorubicin and methotrexate are both transport substrates for *ABCC2*, which caused these variants to be studied, but this does not necessarily explain the causative functional background of the association that is observed. On the other hand, in the pathway approach study by Caronia et al. it was found that per T-allele of the *ABCC3* rs4148416 variant, patients have an eightfold higher risk of death, and 6 times lower risk on event-free survival, and this association was consistently replicated in two other cohorts [33, 38, 39]. *ABCC3* codes for multidrug resistance protein 3 (MRP3) and is an important transporter of bile salts, but is also involved in efflux of methotrexate from liver and kidney cells [90, 91].

The necessity of routine MTX plasma concentration measurement during treatment with MTX has allowed several research groups to study genetic variation involved MTX pharmacokinetics. This was not one of the clinical outcome measures of interest of this systematic review, and therefore these studies were excluded as shown in Fig. 1, however, genetic variants that are associated to high MTX plasma levels may also give increased risk for toxicity and genetic variants that are associated to low MTX plasma levels may also predispose to a suboptimal treatment response. Lui et al*.* found three variants localized in *ABCG2* to be associated with methotrexate clearance in patients with osteosarcoma, namely rs13120400, rs13137622, rs12505410 [31]. Rs13120400 was the most significant variant, and the CC genotype of this variant was previously also associated to increased response to methotrexate in psoriasis patients [92]. In addition, in the study by Hegyi et al*.*, which included 59 osteosarcoma patients, *ABCG2* rs2231142 was found to be significantly associated with a longer half-life time of methotrexate [60]. However, this variant is not in LD with variants identified by Lui et al.. *ABCG2* codes for the breast cancer resistance protein (BCRP) and has an important role in the transport of methotrexate out of the liver and kidney and knockdown of abcg2 increased the bioavailability of methotrexate in mice [91, 93]. Both *ABCC3* and *ABCG2* may be of interest for further investigation with a larger patient cohort to relate it to both pharmacokinetic parameters and clinical outcomes of treatment.

Six variants were found to be associated with doxorubicin-induced cardiotoxicity in a cohort containing osteosarcoma patients and were replicated minimally once in an independent patient cohort, namely *CELF4* rs1786814, *HAS3* rs2232228, *RARG* rs2229774, *SLC22A17* rs4982753, *SLC22A7* rs4149178, *SLC28A3* rs7853758. The associations with *HAS3* rs2232228 and *CELF4* rs1786814 identified by Wang et al*.,* consisted of gene-environment interactions, which means the variant effect is larger in patients that received a higher dose of doxorubicin. This emphasizes the importance of doxorubicin dose in the development of cardiotoxicity and in the effect size of genetic variants. CUGBP Elav-like family member 4 (CELF4) is involved in splicing of *TNNT2,* which codes for cardiac troponin T (cTnT). cTnT plays a role in Ca^2+^ signaling and contraction of the heart muscle and is a biomarker for myocardial damage [94]. Whereas the embryonal *TNNT2* splicing variant, carrying an additional exon 5, is usually downregulated in adults, patients with *CELF4* rs1786814 GG genotype express both the adult and embryonal *TNNT2* splicing variant. This results in a temporally split myofilament response to calcium, decreasing the ventricular pumping efficiency, and thereby increasing the risk on dilated cardiomyopathy and cardiotoxicity [53, 95]. In addition, pathogenic variants in *TNNT2* are an established cause of hypertrophic and dilated cardiomyopathy [96]. Adult and pediatric patients with cancer who developed chemotherapy-induced cardiomyopathy have an increased prevalence of pathogenic variants in sarcomere genes compared to controls, indicating that genetics involved in susceptibility to cardiomyopathy, such as mutations in sarcomere genes, may also be of importance in doxorubicin-induced cardiotoxicity [97]. *HAS3* encodes for hyaluronan which is a component of the extracellular matrix and is involved in tissue remodeling after cardiac damage. In addition, hyaluronan reduces cardiac injury caused by ROS, which is an important element of doxorubicin-induced cardiac damage. Furthermore, the *RARG* gene codes for retinoic acid receptor gamma and binds to the topoisomerase IIβ (Top2b) promotor to repress its expression. Top2b is a target of doxorubicin mediated DNA damage, and if Top2b expression is low in cardiac tissue due to repression by *RARG*, the tissue is less susceptible to damage caused by doxorubicin [48]. Despite that replication studies of the association of the *RARG* variant with cardiotoxicity were inconsistent, a functional study in iPSC-derived cardiomyocytes showed that the variant *RARG* increases sensitivity to doxorubicin-induced cardiomyopathy [98]. Lastly, three variants in genes of the solute carrier transporter family were associated to doxorubicin-induced cardiomyopathy. A variant downstream of *SLC22A17* was associated to cardiotoxicity. *SLC22A17* is ubiquitously expressed, also in the heart, and plays a role in iron transport and homeostasis. Accumulation of iron in mitochondria can cause doxorubicin-induced cardiotoxicity, however the exact role of SLC22A17 in this process is not studied [99]. Secondly, *SLC22A7* encodes for the organic anion transporter 2 (OAT2), which is highly expressed in liver and kidney and is known to play an important role in clearance of medicines but is not previously indicated in transport of cisplatin, doxorubicin or methotrexate. Lastly, a variant in *SLC28A3* was associated with cardiotoxicity. *SLC28A3* codes for the sodium-coupled nucleoside transporter 3 (CNT3). Only for CNT3, it was established to transport doxorubicin, indicating it may play a role in doxorubicin pharmacokinetics [100]. Despite that this was not shown for OAT2, it does have considerable overlap in substrates with CNT3 and transports several nucleoside-based drugs, for example 5-fluorouracil and zidovudine [101]. More recently studies have been performed that used exome and genome-sequencingto identify genes associated with cardiotoxicity [22, 23]. These studies clearly demonstrated that these large scale hypothesis generating studies are of added value to identify new genes linked to cardiotoxicity. These approach in combination with more advanced analysis methods like machine learning will certainly lead to more insights in the genetic background of anthracycline induced cardiotoxicity. Which eventually might lead to prediction models that might be used in the clinical setting as nicely demonstrated by the first attempts of Chaix and colleagues [24].

While cisplatin-induced ototoxicity is one of the most prevalent adverse effects of cisplatin treatment, it was only investigated in one of the studies that were identified in this systematic review [25]. This recent study identified *TCERG1L* to be associated with ototoxicity in a relatively large European cohort of patients with childhood cancer and replication in similar cohorts. As the variant could not be linked to age-related hearing loss or congenital hearing loss, strongly suggesting that the association si specific for cisplatin induced hearing loss. The authors also showed that overexpression of the gene resulted in an increased resistance to cisplatin and could link this to a reduced pro inflammatory cytokine secretion. This inflammatory response is in line with previous methylation studies that linked the gene to colon cancer and inflammatory bowel disease [102–104]. However the exact role of the gene in cisplatin response needs to be investigated in more depth. Although this is a large GWAS on cisplatin induced hearing loss it is advisable to preform GWASes including more patients to confirm the association. GWAS on cisplatin-induced ototoxicity that included patients with osteosarcoma, was performed in 2009 and was therefore excluded from this review [105]. In this study, Ross et al. identified genetic variants in the *TPMT* and *COMT* gene that are associated to cisplatin-induced hearing loss in pediatric patients with cancer. However, replication studies are very contradictive, as shown in a meta-analysis by Thiessen et al. in 2018 [17, 18]. Whereas this was the only GWAS that included patients with osteosarcoma, work from other patient cohorts treated with cisplatin may be applicable to patients with osteosarcoma too. Xu et al. identified that the A-allele of *ACYP2* rs1872328 gives an increased risk to cisplatin-induced ototoxicity in a pediatric brain tumor cohort [14]. In 2020, Clemens et al. showed in a meta-analysis of 5 studies with a total of 1418 pediatric patients that this association was also found in cohorts containing patients with osteosarcoma (OR (95%CI) = 3.94 (1.04–14.93), *p* = 0.04). In addition, they showed a significant association of *SLC22A2* rs316019 with cisplatin induced hearing loss in a meta-analysis of 4 studies (OR (95%CI) = 1.46 (1.07–2.00), *p* = 0.02). Whereas these results are significant, the heterogeneity between studies remained an obstacle (I^2^ = 66% for *ACYP2* rs1872328 and 44% for *SLC22A2* rs316019). In conclusion, in order to find variants associated with cisplatin-induced ototoxicity, the priority would be to perform a GWAS with large patient cohorts to find reliable results. In the meantime, *ACYP2* rs1872328 and *SLC22A2* rs316019 could be studied further to find out their true potential for clinical practice.

In general, the quality of reporting data in the included studies was good, as shown with the quality assessment using STREGA guidelines. However, there was minimal reporting on follow-up and missing data. All data in the studies was collected retrospectively and there was no prospective follow-up and, therefore, it is sensible that nothing was reported on patients that were lost to follow-up. It was also not reported how missing data was handled in statistical analysis or what kind of analysis was used for the main comparison regarding the missing data. However, it is assumed that these studies did a complete case analysis, because that is most conventional in retrospective genetic association studies. Therefore, this does not compromise the quality of these articles.

The aim of this review was to identify variants that were discovered to be associated to a phenotype in a hypothesis-free manner and might form the basis for future pharmacogenomic studies in osteosarcoma. The most evident method to identify articles that describe these variants would be to limit the systematic search to GWASs and large scale sequencing studies, as they are the textbook example to hypothesis-free research. However, the number of such studies is limited in pediatric oncology cohorts and even more so in osteosarcoma cohorts. In addition, when a broad range of ADME genes is studied, it does not restrict itself to only genes that are previously indicated in the pathway of a drug. In order to include all literature with a hypothesis-free component, a boundary has been set to articles that assess more than 10 variants in more than 5 genes. The smallest studies in this review, by Hattinger et al., Hegyi et al., and Windsor et al. (45, 29 and 36 variants in cohorts of 57, 59 and 58 osteosarcoma patients), showed the poorest reproducibility (Table S8, S9, S10) [35, 37, 60]. Possibly, due to the small number of patients in these studies, the power was too low to perform multiple testing correction causing the authors to report false-positive findings.

Not only in the case of small studies, but also in larger studies, replication remains laborious. Our structured search shows that many replication studies have been performed but the majority do not confirm the findings from the discovery study with significant results (see Fig. 2). Obvious explanations for this are the general heterogeneity between cohorts, different ethnicities, phenotypes and treatment regimens. An important source for heterogeneity might be co-medication. For instance use of otoprotective agents might interfere with genetic studies on ototoxicity. Unfortunately, information on co-medication is often incomplete or not completely reported. To minimize heterogeneity in genetic studies it is advisable to keep track of the co-medication used during treatment. In addition, power in discovery studies is often too low to correct for multiple testing, leading to false-positive findings. Insufficient power in replication studies may also stand in the way of confirming true-positive findings. As a solution for that, meta-analysis of the discovery and replication cohorts could increase the total power, however in this review there was too much heterogeneity between studies to perform reliable meta-analyses. To study treatment response different outcome parameters, such as overall survival, event-free survival, disease-free survival, progression-free survival, histological response and tumor necrosis were used, making it impossible to combine in a meta-analysis. For toxicity outcomes, there were large differences in grading systems and the exact definitions for which patients are considered cases. In pediatric patient cohorts, cardiotoxicity is defined in as fractional shortening below a limit that varies among studies. In adults, cardiotoxicity is defined as a decrease of ejection fraction below the lower limit of normal or a large absolute reduction in ejection fraction, making it impossible to combine pediatric and adult patient cohorts in meta-analysis. The introduction of more sensitive imaging tools may allow for earlier detection of cardiac dysfunction and homogenize phenotypes, for example using global longitudinal strain (GLS), 3D volumetric echocardiographic or MRI. Lastly, confidence intervals of discovery and replication studies in this review do not always overlap, and therefore, it is already to be predicted that meta-analyses will be very heterogenous.

Although the studies included in this review do give insights in the genes that might be involved in treatment response and toxicity in osteosarcoma we are still far from clinical implementation. As shown in this review many results are hard to confirm and most studies only investigated a limited set of genes whereas it is to be expected that a combination of several genes and environmental factors will be necessary to fully predict treatment outcome. In the future, the consistency of phenotypes would be improved if research groups with similar interests would collaborate to clearly define the phenotypes together this will make the studies less heterogeneous. Besides these collaborations will allow combination of cohorts to enlarge the patient populations investigated which will give more power to the studies. Although combining patients with childhood cancer that are treated in a similar manner is a good approach to increase patient populations it might also be of added value when analysing homogenous patient groups (e.g. with one type of cancer and the same treatment protocol) as this might give valuable information on differences between patient groups. To be able to perform such analysis in the future it might be advisable to follow patients with childhood cancer from the beginning of their disease and treatment and collect treatment outcome and toxicities in a structured manner as part of clinical care. Such prospectively collected data will be a great source for future genetic studies.

## Conclusion

To conclude, in this systematic review, twenty articles were found that aimed to identify novel genetic variants involved in treatment toxicity or treatment response in patients with osteosarcoma. Most research was done on doxorubicin-induced cardiomyopathy and for seven genetic variants *in CELF4, GPR35, HAS3, RARG, SLC22A17, SLC22A7* and *SLC28A3*, replication studies were performed without consistent results. Genetic variants *in ABCC3, ABCC5, FasL, GLDC* and *GSTP1* were repeatedly associated to osteosarcoma treatment outcome, using very heterogeneous efficacy outcomes. Studies reporting on bone marrow, nephro- and/or hepatotoxicity were small and had poor reproducibility. Moreover, only one article assessed cisplatin-induced ototoxicity. Despite that these results are promising and may have great potential for the future, replications often remain contradictory. Therefore, hypothesis-generating studies in large patient cohorts will be necessary to confirm these variants and to discover novel associations. Large initiatives, for example Euramos-1 or the Children’s Oncology Group, could liaise with other research groups around the world with similar interests to boost the discovery of pharmacogenetic variants. Thereafter, functional studies are important to elucidate the mechanism behind the association and, ultimately, interventions should be established that make use of these associations to give patients with osteosarcoma a treatment that fits the needs of the individual best. 

## Supplementary Information

**Additional file 1:** **Figure S1.** General search strategy for electronic databases. **Table S1.** Search strategy for electronic databases. **Table S2.** Inventory of number of genes studied by the 98 genetic association studies. The bold line indicates the border between studies that are excluded (above line) or included (below line). **Table S3.** Inventory of number of variants studied by the 98 genetic association studies. The bold line indicates the border between studies that are excluded (above line) or included (below line). **Table S4.** Publications that were not included in this systematic review. **Table S5.** Quality assessment form. **Table S6.** Results of quality assessment according to the STrengthening the REporting of Genetic Association studies (STREGA) guidelines for reporting of genetic association studies was adjusted to be more applicable to pharmacogenetic studies. **Table S7.** Results question 5 of the quality assessment regarding to reporting of relevant baseline characteristics. Characteristics were considered relevant if they were reported in 2 or more studies.

**Additional file 2:** **Table S8.** Characteristics and results of independent discovery and replication cohorts, studying genetic variants associated with treatment response in patients with osteosarcoma. **Table S9.** Characteristics and results of independent discovery and replication cohorts, studying genetic variants associated with doxorubicin-induced cardiotoxicity. **Table S10.** Characteristics and results of independent discovery and replication cohorts, studying genetic variants associated with bone marrow- hepato- and nephrotoxicity after treatment with cisplatin, doxorubicin or methotrexate.

## Data Availability

All relevant information on data generated or analyzed during this study are included in this published article and its supplementary information files. Other intermediate datasets are available from the corresponding author on reasonable request.

## Abbreviations

MAP: Methotrexate, adriamycin (doxorubicin), cisplatin chemotherapy regimen
GWAS: Genome-wide association study
MeSH: Medical subject headings
STREGA: STrengthening the REporting of Genetic Association
MTX: Methotrexate
ADME: Absorption, distribution, metabolism and excretion
PFS: Progression free survival
EFS: Event free survival
OS: Overall survival
CYP: Cytochrome P450
