# Supplementary material for: Pharmacogenetics of chemotherapy treatment response and -toxicities in patients with osteosarcoma: a systematic review
Source: BMC Cancer. 2022 Dec 19;22:1326. doi: 10.1186/s12885-022-10434-5 (PMC9761983; doi:10.1186/s12885-022-10434-5)
Supplement: Supplementary file 1 — Additional file 1: Figure S1. General search strategy for electronic databases. Table S1. Search strategy for electronic databases. Table S2. Inventory of number of genes studied by the 98 genetic association studies. The bold line indicates the border between studies that are excluded (above line) or included (below line). Table S3. Inventory of number of variants studied by the 98 genetic association studies. The bold line indicates the border between studies that are excluded (above line) or included (below line). Table S4. Publications that were not included in this systematic review. Table S5. Quality assessment form. Table S6. Results of quality assessment according to the STrengthening the REporting of Genetic Association studies (STREGA) guidelines for reporting of genetic association studies was adjusted to be more applicable to pharmacogenetic studies. Table S7. Results question 5 of the quality assessment regarding to reporting of relevant baseline characteristics. Characteristics were considered relevant if they were reported in 2 or more studies. [file 12885_2022_10434_MOESM1_ESM.docx]

**File S1. Literature Search and quality assessment**

Figure S1. General search strategy for electronic databases.

**Osteosarcoma**

Childhood cancer

AND

Cisplatin OR doxorubicin OR MTX

AND

Toxicity

GWAS

OR

Genetic variation

Treatment outcome

OR

Toxicity

OR

Cisplatin OR doxorubicin OR MTX

**OR**

**AND**

**AND**

Table S1. Search strategy for electronic databases

| **Search source** | Pubmed/MEDLINE and Embase |
| --- | --- |
| **Search date** | 30^th^ of September 2020, updated on 19^th^ of July 2022 |
| **Search restrictions** | None |
| **Filters used** | None |
| **Pubmed/MEDLINE search strategy** | ((“Osteosarcoma”[tiab] OR "osteosarcoma"[MeSH Terms] OR “Osteosarcomas”[tiab] OR “Sarcoma, Osteogenic”[tiab] OR “Sarcomas, Osteogenic”[tiab] OR “Osteogenic Sarcomas”[tiab] OR “Osteogenic Sarcoma”[tiab] OR "Bone Neoplasms/drug therapy"[Mesh] OR “bone cancer”[tiab] OR “bone tumor”[tiab] or “bone tumour”[tiab] OR “bone tumors”[tiab] or “bone tumours”[tiab]) OR (("Child"[Mesh] OR "Adolescent”[Mesh] OR childhood cancer*[tiab] OR adolescent cancer*[tiab] OR pediatric cancer*[tiab] OR childhood tumor*[tiab] OR childhood tumour*[tiab]) AND ("Methotrexate"[Mesh] OR “Methotrexate”[tiab] OR "Cisplatin"[Mesh] OR “Cisplatin”[tiab] OR “NSC-119875”[tiab] OR “cis-Platinum”[tiab] OR “cis Platinum”[tiab] OR “CDDP”[tiab] OR "Doxorubicin"[Mesh] OR “Doxorubicin”[tiab] OR "Anthracyclines"[Mesh:NoExp] OR “anthracycline”[tiab] OR “anthracyclines”[tiab] AND ("Ototoxicity"[Mesh] OR "Hearing Loss/chemically induced"[Mesh] OR "Hearing Loss/genetics"[Mesh] OR "Hearing Loss, Sensorineural"[Mesh] OR "Ear Diseases/chemically induced"[Mesh] OR "Ear Diseases/genetics"[Mesh] OR ototoxic*[tiab] OR “hearing loss”[tiab] OR "Renal Insufficiency"[Mesh] OR "Kidney Diseases/chemically induced"[Mesh] OR "Kidney Diseases/drug effects"[Mesh] OR "Kidney Diseases/genetics"[Mesh] OR "Acute Kidney Injury/chemically induced"[Mesh] OR "Acute Kidney Injury/drug effects"[Mesh] OR "Acute Kidney Injury/genetics"[Mesh] OR nephrotoxic*[tiab] OR “renal failure”[tiab] OR “kidney failure”[tiab] OR “renal function”[tiab] OR "Heart Diseases/chemically induced"[Mesh] OR "Heart Diseases/genetics"[Mesh] OR "Cardiomyopathies/chemically induced"[Mesh] OR "Cardiomyopathies/genetics"[Mesh] OR "Cardiotoxicity"[Mesh] OR cardiac toxic*[tiab] OR cardiotoxic*[tiab] OR heart toxic*[tiab] OR “cardiomyopathy”[tiab] or "Bone Marrow/drug effects"[Mesh] OR "Hematopoiesis/drug effects"[Mesh] OR "Hematopoiesis/genetics"[Mesh] OR "Bone Marrow Cells/drug effects"[Mesh] OR “myelosuppression”[tiab] OR “myelosuppressive toxicity”[tiab] OR hematologic toxic*[tiab] OR ”anemia”[tiab] OR “myelopoiesis”[tiab] OR "Chemical and Drug Induced Liver Injury"[Mesh] OR "Liver Diseases/chemically induced"[Mesh] OR "Liver Diseases/drug effects"[Mesh] OR "Liver/drug effects"[MAJR] OR "Liver Diseases/genetics"[Mesh] OR “hepatotoxicity”[tiab] OR liver toxic*[tiab] OR hepatotoxic*[tiab])))) AND (("Genome-Wide Association Study"[Mesh] OR “GWAS”[tiab] OR “Genome-wide association study”[tiab] OR “Genome-Wide Association Studies”[tiab] OR “Whole Genome Association Analysis”[tiab] OR “GWA Study”[tiab] OR “GWA Studies”[tiab] OR “Genome Wide Association Scan”[tiab] OR “Genome Wide Association Studies”[tiab] OR “Genome Wide Association Study”[tiab] OR “Genome Wide Association Analysis”[tiab] OR “Whole Genome Association Study”[tiab] OR “Genome-wide scan”[tiab] OR “Genome wide scan”[tiab] OR “Genome-wide association analysis”[tiab] OR “Genome-wide association analysis”[tiab]) OR ("Genomic Structural Variation"[Mesh] OR "Pharmacogenetics"[Mesh] OR "Polymorphism, Genetic"[Mesh] OR "Polymorphism, Single Nucleotide"[Mesh] OR "Genotype"[Mesh] OR "Germ-Line Mutation"[Mesh] OR genetic variant*[tiab] OR genetic variation*[tiab] OR gene variation*[tiab] OR gene variant*[tiab] OR polymorphism*[tiab] OR SNP[tiab] OR pharmacogenetic*[tiab] OR pharmacogenomic*[tiab] OR genotype*[tiab] OR variation*[tiab] OR variant*[tiab] OR genetic variability[tiab] OR germ-line mutation*[tiab] OR germ-line variation*[tiab])) AND ("Doxorubicin/adverse effects"[Mesh] OR "Doxorubicin/toxicity"[Mesh] OR "Doxorubicin/therapeutic use"[Mesh] OR "Doxorubicin/metabolism"[Mesh] OR "Doxorubicin/pharmacokinetics"[Mesh] OR “Doxorubicin”[tiab] OR "Anthracyclines/adverse effects"[Majr] OR "Anthracyclines/toxicity"[Majr] OR "Anthracyclines/therapeutic use"[Majr] OR "Anthracyclines/metabolism"[Majr] OR "Anthracyclines/pharmacokinetics"[Majr] OR “anthracycline”[tiab] OR “anthracyclines”[tiab] OR "Cisplatin/adverse effects"[Mesh] OR "Cisplatin/metabolism"[Mesh] OR "Cisplatin/pharmacokinetics"[Mesh] OR “Cisplatin”[tiab] OR “NSC-119875”[tiab] OR “cis-Platinum”[tiab] OR “cis Platinum”[tiab] OR “CDDP”[tiab] OR "Methotrexate/adverse effects"[Mesh] OR "Methotrexate/toxicity"[Mesh] OR "Methotrexate/therapeutic use"[Mesh]OR "Methotrexate/metabolism"[Mesh] OR "Methotrexate/pharmacokinetics"[Mesh] OR “Methotrexate”[tiab] OR "Ototoxicity"[Mesh] OR "Hearing Loss/chemically induced"[Mesh] OR "Hearing Loss/genetics"[Mesh] OR "Hearing Loss, Sensorineural"[Mesh] OR "Ear Diseases/chemically induced"[Mesh] OR "Ear Diseases/genetics"[Mesh] OR ototoxic*[tiab] OR “hearing loss”[tiab] OR "Renal Insufficiency"[Mesh] OR "Kidney Diseases/chemically induced"[Mesh] OR "Kidney Diseases/drug effects"[Mesh] OR "Kidney Diseases/genetics"[Mesh] OR "Acute Kidney Injury/chemically induced"[Mesh] OR "Acute Kidney Injury/drug effects"[Mesh] OR "Acute Kidney Injury/genetics"[Mesh] OR nephrotoxic*[tiab] OR “renal failure”[tiab] OR “kidney failure”[tiab] OR “renal function”[tiab] OR "Heart Diseases/chemically induced"[Mesh] OR "Heart Diseases/genetics"[Mesh] OR "Cardiomyopathies/chemically induced"[Mesh] OR "Cardiomyopathies/genetics"[Mesh] OR "Cardiotoxicity"[Mesh] OR cardiac toxic*[tiab] OR cardiotoxic*[tiab] OR heart toxic*[tiab] OR “cardiomyopathy”[tiab] or "Bone Marrow/drug effects"[Mesh] OR "Hematopoiesis/drug effects"[Mesh] OR "Hematopoiesis/genetics"[Mesh] OR "Bone Marrow Cells/drug effects"[Mesh] OR “myelosuppression”[tiab] OR “myelosuppressive toxicity”[tiab] OR hematologic toxic*[tiab] OR ”anemia”[tiab] OR “myelopoiesis”[tiab] OR "Chemical and Drug Induced Liver Injury"[Mesh] OR "Liver Diseases/chemically induced"[Mesh] OR "Liver Diseases/drug effects"[Mesh] OR "Liver/drug effects"[MAJR] OR "Liver Diseases/genetics"[Mesh] OR “hepatotoxicity”[tiab] OR liver toxic*[tiab] OR hepatotoxic*[tiab] OR "Treatment Outcome"[Mesh] OR “Efficacy”[tiab] OR “Treatment efficacy”[tiab] OR "Survival Analysis"[Mesh] OR “Survival”[tiab] OR “Overall survival”[tiab] OR "Disease Progression"[Mesh] OR “Progression”[tiab] OR “Progressive disease” OR “disease, progressive”[tiab] OR "Recurrence"[Mesh] OR "Neoplasm Recurrence, Local"[Mesh] OR “Recurrence“[tiab] OR “Recurrences”[tiab] OR “Relapse”[tiab] OR “Relapses”[tiab] OR "Prognosis"[Mesh] OR “prognosis”[tiab] OR "Neoplasm Metastasis"[Mesh] OR “metastasis”[tiab]) |
| **Embase search strategy** | ((exp osteosarcoma/ or osteosarcoma/dt or bone tumor/dt, si or (osteosarcoma* or osteogenic sarcoma* or bone cancer* or bone tumor* or bone tumour*).ti,ab,kw. ) or ((juvenile/ or adolescent/ or child/ or childhood cancer.mp. or exp childhood cancer/) and (methotrexate/ae, dt, to, pd or cisplatin/ae, dt, to, pd or doxorubicin/ae, dt, to, pd or anthracycline antibiotic agent/ae, dt, to, pd or (cisplatin or doxorubicin or methotrexate or MTX or anthracycline).ti,ab,kw.) and (exp ototoxicity/si or ototoxicity.mp. or high frequency hearing loss/si or exp blood toxicity/co, si or exp bone marrow toxicity/co, si or exp bone marrow suppression/co, si or blood toxicity.mp. or bone marrow toxicity.mp. or bone marrow suppression.mp. or anemia/si or infection/si or leukopenia/si or exp cardiotoxicity/co, si or cardiotoxicity.mp. or cardiomyopathy/co, si or cardiovascular disease/co, si or exp liver toxicity/ or liver toxicity.mp. or hepatotoxicity.mp. or liver injury/co, si or alanine aminotransferase/ or alanine aminotransferase blood level/ or exp nephrotoxicity/ or nephrotoxicity.mp. or creatinine blood level/ or creatinine/ or *"pharmacokinetic parameters"/ or *"pharmacogenetics"/ or pharmacokinetics/ or drug absorption/ or drug clearance/ or drug distribution/ or drug elimination/ or drug excretion/ or drug metabolism/ or (ototoxic* or "hearing loss" or blood toxicity or bone marrow toxic* or bone marrow suppression or cardiotoxic* or cardiomyopathy or liver toxic* or hepatotoxic* or nephrotoxic* or kidney toxic*).ti,ab,kw.))) AND (exp genetic association/ or exp genome-wide association study/ or exp single nucleotide polymorphism/ or pharmacogenetics.mp. or exp pharmacogenetics/ or exp genetic variability/ or exp genetic variation/ or exp genotype/ or genotype.mp. or exp genetic polymorphism/) AND (treatment outcome/ or clinical outcome/ or disease free interval/ or disease worsening with drug treatment/ or outcome assessment/ or outcomes research/ or partial drug response/ or treatment failure/ or exp survival/ or relapse/dr, dt, si or exp recurrent disease/dr, dt, si or (exp ototoxicity/si or ototoxicity.mp. or high frequency hearing loss/si or exp blood toxicity/co, si or exp bone marrow toxicity/co, si or exp bone marrow suppression/co, si or blood toxicity.mp. or bone marrow toxicity.mp. or bone marrow suppression.mp. or anemia/si or infection/si or leukopenia/si or exp cardiotoxicity/co, si or cardiotoxicity.mp. or cardiomyopathy/co, si or cardiovascular disease/co, si or exp liver toxicity/ or liver toxicity.mp. or hepatotoxicity.mp. or liver injury/co, si or alanine aminotransferase/ or alanine aminotransferase blood level/ or exp nephrotoxicity/ or nephrotoxicity.mp. or creatinine blood level/ or creatinine/ or (ototoxic* or "hearing loss" or blood toxicity or bone marrow toxic* or immunotoxic* or lymphocytotoxic* or bone marrow suppression or cardiotoxic* or cardiomyopathy or liver toxic* or hepatotoxic* or nephrotoxic* or kidney toxic*).ti,ab,kw.) or (methotrexate/ae, dt, to, pd or cisplatin/ae, dt, to, pd or doxorubicin/ae, dt, to, pd or anthracycline antibiotic agent/ae, dt, to, pd) or (cisplatin or doxorubicin or methotrexate or MTX or anthracycline).ti,ab,kw.) |

Table S2. Inventory of number of genes studied by the 98 genetic association studies. The bold line indicates the border between studies that are excluded (above line) or included (below line)

| Number of genes | Number of publications |
| --- | --- |
| 1 | 31 |
| 2 | 11 |
| 3 | 13 |
| 4 | 7 |
| 5 | 7 |
| 6 | 1 |
| 8 | 2 |
| 9 | 2 |
| 10 | 1 |
| 11 | 1 |
| 12 | 1 |
| 15 | 2 |
| 21 | 1 |
| 24 | 1 |
| 26 | 1 |
| 31 | 1 |
| 54 | 1 |
| 220 | 1 |
| 231 | 1 |
| 300 | 1 |
| 2100 | 1 |
| 20000 (genome-wide) | 10 |

Table S3. Inventory of number of variants studied by the 98 genetic association studies. The bold line indicates the border between studies that are excluded (above line) or included (below line)

| Number of variants | Number of publications |
| --- | --- |
| 1-5 | 57 |
| 6-10 | 13 |
| 11-15 | 4 |
| 16-20 | 2 |
| 21-25 | 2 |
| 26-30 | 2 |
| 31-35 | 0 |
| 36-40 | 1 |
| 41-45 | 2 |
| 46-65 | 0 |
| 65-70 | 1 |
| >100 | 2 |
| >1000 | 4 |
| >100000 | 10 |

Table S4. Publications that were excluded from the systematic review in the last phases of the selection as a result of the pre-defined inclusion criteria.

| Author | Year | Title | Exclusion phase | Exclusion reason | Ref. |
| --- | --- | --- | --- | --- | --- |
| Clemens *et al*. | 2020 | Genetic variation of cisplatin-induced ototoxicity in non-cranial-irradiated pediatric patients using a candidate gene approach: The International PanCareLIFE Study | Inventory # variants and # genes (Table S3 and S4) | ≤10 variants but >5 genes | [1] |
| Feng *et al.* | 2022 | The effects of common variants in MDM2 and GNRH2 genes on the risk and survival of osteosarcoma in Han populations from Northwest China | Inventory # variants and # genes (Table S3 and S4) | ≤5 genes but >10 variants | [2] |
| Goricar *et al*. | 2014 | Influence of the folate pathway and transporter polymorphisms on methotrexate Treatment outcome in osteosarcoma | Inventory # variants and # genes (Table S3 and S4) | ≤10 variants but >5 genes | [3] |
| Goricar *et al*. | 2015 | Genetic variability of DNA repair mechanisms and glutathione-S-transferase genes influences treatment outcome in osteosarcoma | Inventory # variants and # genes (Table S3 and S4) | ≤10 variants but >5 genes | [4] |
| Kang *et al*. | 2019 | Relationship of common variants in Interleukin 33 gene with susceptibility and prognosis of osteosarcoma in Han Chinese population | Inventory # variants and # genes (Table S3 and S4) | ≤5 genes but >10 variants | [5] |
| Langer *et al*. | 2020 | Usefulness of current candidate genetic markers to identify childhood cancer patients at risk for platinum-induced ototoxicity: Results of the European PanCareLIFE cohort study | Final full text assessment | Replication study | [6] |
| Lanvers-Kaminsky *et al*. | 2015 | Human OCT2 variant c.808G>T confers protection effect against cisplatin-induced ototoxicity | Inventory # variants and # genes (Table S3 and S4) | ≤5 genes but >10 variants | [7] |
| Lui *et al*. | 2018 | A Pharmacokinetic and Pharmacogenetic Analysis of Osteosarcoma Patients Treated With High-Dose Methotrexate: Data From the OS2006/Sarcoma-09 Trial | Final full text assessment | Wrong outcome | [8] |
| Ruiz-Pinto *et al*. | 2018 | Exome array analysis identifies ETFB as a novel susceptibility gene for anthracycline-induced cardiotoxicity in cancer patients | Final full text assessment | No osteosarcoma in discovery phase | [9] |
| Sági *et al*. | 2018 | Possible roles of genetic variations in chemotherapy related cardiotoxicity in pediatric acute lymphoblastic leukemia and osteosarcoma | Final full text assessment | No osteosarcoma in discovery phase | [10] |
| Spracklen *et al*. | 2014 | Genetic variation in otos is associated with cisplatin-induced ototoxicity | Inventory # variants and # genes (Table S3 and S4) | ≤5 genes but >10 variants | [11] |
| Sun *et al*. | 2015 | Genetic polymorphisms in nucleotide excision repair pathway influences response to chemotherapy and overall survival in osteosarcoma | Inventory # variants and # genes (Table S3 and S4) | ≤10 variants but >5 genes | [12] |
| Vargas-Neri *et al.* | 2022 | Pharmacogenomic study of anthracycline-induced cardiotoxicity in Mexican pediatric patients | Inventory # variants and # genes (Table S3 and S4) | ≤10 variants and ≤5 genes | [13] |
| Visscher *et al*. | 2013 | Validation of variants in SLC28A3 and UGT1A6 as genetic markers predictive of anthracycline-induced cardiotoxicity in children | Final full text assessment | Replication study | [14] |

Table S5. Quality assessment form.

QUALITY ASSESMENT FORM

*Study rationale*

1. **Was there a clear rationale for the selected candidate genes?**Yes; No; n/a (for GWAS)

*Sample selection*

1. **Was a power calculation performed?**
   Yes, before initiation of study; Yes, but retrospectively; No
2. **If a power calculation was performed (Yes to #2), were the number of inclusions required for a power of 80 met?**Yes; No; n/a (No to #2)
3. **Were in- and exclusion criteria clearly described?**Yes; No
4. **Were sufficient basic characteristics reported for the study population?***

Yes; No, name characteristics

1. **Was a validation cohort selected (in this study) to test reproducibility of findings in the original cohort?**Yes; No

** The basic characteristics that were reported in 2 or more studies were considered as most relevant baseline characteristics. Based on that and depending on the outcome variable, an estimation was made if this article reported sufficient baseline characteristics.*

*Treatment and outcome*

1. **Were all patients treated according to label?**Yes; No; NR
2. **Were clinical data collected prospectively or retrospectively?**

Prospectively; Retrospectively; NR

*Follow-up and missing data*

1. **Does the study describe the number of participants who withdrew and/or were lost to follow‐up after the start of the observation period?**Yes, complete (could replicate); Yes, partial (could not replicate); No; n/a
2. **What was the overall percentage of participants who withdrew or were lost to follow‐up after the start of the observation period?**Specify percentage; NR; n/a
3. **What was the overall percentage of missing data for the association between the pharmacogenetics interaction and the main outcome?**Specify percentage; NR; n/a

*Genotyping*

1. **Specify genotyping method(s) used.**
   PCR; Sanger sequencing; allelic discrimination assays; GWA array; other (specify); NR
2. **For studies using genome‐wide association study (GWAS) data, specify the allele-calling algorithm used.**Specify algorithm; NR; n/a
3. **How many SNPs were excluded based on call rate, and what cut-off point for exclusion was used?**Specify number of excluded SNPs (specify cut-off point); NR
4. **Was duplicate genotyping performed?**Yes; No; NR; n/a (for GWAS)
5. **If duplicate genotyping was performed (Yes to #16), what was the duplicate genotyping concordance?**
   Specify percentage; n/a
6. **Was Hardy-Weinberg equilibrium calculated?**Yes; No
7. **If alleles were not in HWE, how were they handled?**Excluded based on *P*-value; Included (specify); Other (specify)

*Statistical analyses*

1. **How were missing data handled (select all that apply)?**Excluded (complete case analysis)

Single imputatation (specify method)
Multiple imputation (specify method)
Other (specify)
NR
n/a

1. **What type of analysis was used for the main comparison?**Intention to treat analysis
   As treated/complete case analysis
   Other (specify)
   NR
2. **For studies investigating more than one genetic variant, was adjustment for multiple testing applied?**Yes; No; n/a
3. **Were analyses performed to assess the effect of confounders?**Yes (specify); No; n/a

*Other*

1. **Please provide any additional remarks (either positive or negative) on quality.**

|  |
| --- |

Table S6. Results of quality assessment according to the STrengthening the REporting of Genetic Association studies (STREGA) guidelines for reporting of genetic association studies was adjusted to be more applicable to pharmacogenetic studies

|  | **Study rationale** |
| --- | --- |
| **Question** | **1. Was there a clear rationale for the selected candidate genes?** |
| Aminkeng *et al.*, 2015 | n/a |
| Bhuvaneshwar *et al.* 2019 | n/a |
| Caronia *et al*., 2011 | n/a |
| Chaix *et al.* 2020 | n/a |
| Gong *et al., 2021* | Yes |
| Hagleitner *et al*., 2015 | Yes |
| Hattinger *et al*. 2016 | Yes |
| Hegyi *et al*., 2017 | Yes |
| Hildebrandt *et al*. 2017 | Yes |
| Hurkmans *et al*., 2020 | Yes |
| Koster *et al*., 2018 | n/a |
| Meijer *et al., 2021* | n/a |
| Ruiz-Pinto *et al.*, 2017 | n/a |
| Sapkota *et al. 2021* | n/a |
| Sapkota *et al. 2022* | n/a |
| Visscher *et al.*, 2012 | Yes |
| Visscher *et al*., 2015 | Yes |
| Wang *et al*., 2014 | Yes |
| Wang *et al*., 2016 | n/a |
| Windsor *et al*., 2012 | Yes |
|  |  |
|  | **Sample selection** |
| **Question** | **2. Was a power calculation performed?** |
| Aminkeng *et al.*, 2015 | No |
| Bhuvaneshwar *et al.* 2019 | No |
| Caronia *et al*., 2011 | No |
| Chaix *et al.* 2020 | No |
| Gong *et al., 2021* | No |
| Hagleitner *et al*., 2015 | Yes, but retrospectively |
| Hattinger *et al*. 2016 | Yes, but retrospectively |
| Hegyi *et al*., 2017 | No |
| Hildebrandt *et al*. 2017 | No |
| Hurkmans *et al*., 2020 | No |
| Koster *et al*., 2018 | No |
| Meijer *et al.* 2021 | No |
| Ruiz-Pinto *et al.*, 2017 | No |
| Sapkota *et al. 2021* | No |
| Sapkota *et al. 2022* | No |
| Visscher *et al.*, 2012 | No |
| Visscher *et al*., 2015 | No |
| Wang *et al*., 2014 | No |
| Wang *et al*., 2016 | Yes, but retrospectively |
| Windsor *et al*., 2012 | No |
|  |  |
| **Question** | **3. If a power calculation was performed (Yes to #2), were the number of inclusions required for a power of 80 met?** |
| Aminkeng *et al.*, 2015 | n/a |
| Bhuvaneshwar *et al.* 2019 | n/a |
| Caronia *et al*., 2011 | n/a |
| Chaix *et al.* 2020 | n/a |
| Gong *et al., 2021* | n/a |
| Hagleitner *et al*., 2015 | Yes |
| Hattinger *et al*. 2016 | No |
| Hegyi *et al*., 2017 | n/a |
| Hildebrandt *et al*. 2017 | n/a |
| Hurkmans *et al*., 2020 | n/a |
| Koster *et al*., 2018 | n/a |
| Meijer *et al.* 2021 | n/a |
| Ruiz-Pinto *et al.*, 2017 | n/a |
| Sapkota *et al. 2021* | n/a |
| Sapkota *et al. 2022* | n/a |
| Visscher *et al.*, 2012 | n/a |
| Visscher *et al*., 2015 | n/a |
| Wang *et al*., 2014 | n/a |
| Wang *et al*., 2016 | Yes |
| Windsor *et al*., 2012 | n/a |
|  |  |
| **Question** | **4. Were in- and exclusion criteria clearly described?** |
| Aminkeng *et al.*, 2015 | Yes |
| Bhuvaneshwar *et al.* 2019 | Yes |
| Caronia *et al*., 2011 | Yes |
| Chaix *et al.* 2020 | Yes |
| Gong *et al., 2021* | Yes |
| Hagleitner *et al*., 2015 | Yes |
| Hattinger *et al*. 2016 | Yes |
| Hegyi *et al*., 2017 | Yes |
| Hildebrandt *et al*. 2017 | Yes |
| Hurkmans *et al*., 2020 | Yes |
| Koster *et al*., 2018 | No |
| Meijer *et al.* 2021 | Yes |
| Ruiz-Pinto *et al.*, 2017 | Yes |
| Sapkota *et al. 2021* | Yes |
| Sapkota *et al. 2022* | Yes |
| Visscher *et al.*, 2012 | Yes |
| Visscher *et al*., 2015 | Yes |
| Wang *et al*., 2014 | Yes |
| Wang *et al*., 2016 | Yes |
| Windsor *et al*., 2012 | Yes |
|  |  |
| **Question** | **5. Were sufficient basic characteristics reported for the study population?** |
| Aminkeng *et al.*, 2015 | Yes; age, gender, cumulative anthracycline exposure, anthracycline chemotherapy, primary diagnosis, radiotherapy involving heart, use of cardioprotectants, duration follow-up |
| Bhuvaneshwar *et al.* 2019 | No |
| Caronia *et al*., 2011 | Yes; age, sex, tumor location, response to treatment, metastasis, survival status, relapse |
| Chaix *et al.* 2020 | Yes, age, cumulative anthracycline dose, dexrazoxane use, radiation therapy involving the heart, cancer diagnosis, LVEF at last follow-up, time from first anthracycline dose to last follow-up echocardiogram, duration of treatment |
| Gong *et al., 2021* | Yes; age, gender, diagnosis, weight, MTX dose |
| Hagleitner *et al*., 2015 | Yes; age, gender, primary metastasis, tumor in axial skeleton, poor histologic response, 5-year PFS |
| Hattinger *et al*. 2016 | Yes; age, gender, tumor site, metastasis at diagnosis, histologic subtype, surgery, treatment |
| Hegyi *et al*., 2017 | Yes; age, gender, risk group, hepatotoxicity, myelotoxicity, peak MTX, 48h MTX, AUX, T1/2 |
| Hildebrandt *et al*. 2017 | Yes; age, gender, race, chest radiation, cancer site, anthracycline cumulative dose, follow-up time, average EF, risk score, hypertension |
| Hurkmans *et al*., 2020 | Yes; age, sex, ethnicity, metastasis, treatment protocol, MTX cumulative dose |
| Koster *et al*., 2018 | Yes; age, sex, vital status, metastasis at diagnosis |
| Meijer *et al.* 2021 | Yes; age, year of diagnosis, time to follow-up, age at follow-up, total cumulative cisplatin dose, sex, tumor type, additional carboplatin |
| Ruiz-Pinto *et al.*, 2017 | Yes; age, sex, primary diagnosis, family history of cardiovascular disease, radiotherapy involving the heart, cumulative anthracyline dose, anthracycline type, concomitant therapy, follow-up |
| Sapkota *et al. 2021* | Yes, age, age at last follow-up, ejection fraction, sex, cardiomyopathy, anthracycline dose, heart radiation dose |
| Sapkota *et al. 2022* | Yes, age at diagnosis, age at last follow-up, ejection fraction, time from diagnosis to echocardiogram showing lowest EF, age at cancer induced cardiac dysfunction, sex, anthracycline dose, heart radiation dose. |
| Visscher *et al.*, 2012 | Yes; age, sex, dose, anthracycline type, tumor type, radiotherapy involving heart, follow-up |
| Visscher *et al*., 2015 | No |
| Wang *et al*., 2014 | Yes; race, age, sex, diagnosis, year of diagnosis, follow-up, cumulative anthracycline dose, chest radiation, age at cardiomyopathy diagnosis, ejection fraction |
| Wang *et al*., 2016 | Yes; race, age at primary cancer, age at study participation, sex, primary diagnosis, year of primary diagnosis, follow-up, chest radiation, ejection fraction, fractional shortening |
| Windsor *et al*., 2012 | Yes; age, follow-up, sex, ethnic group, primary tumor site, metastasis at diagnosis, histological subtype, histological response, death, relapse |
|  |  |
| **Question** | **6. Was a validation cohort selected (in this study) to test reproducibility of findings in the original cohort?** |
| Aminkeng *et al.*, 2015 | Yes |
| Bhuvaneshwar *et al.* 2019 | No |
| Caronia *et al*., 2011 | No |
| Chaix *et al.* 2020 | Yes |
| Gong *et al., 2021* | No |
| Hagleitner *et al*., 2015 | Yes |
| Hattinger *et al*. 2016 | No |
| Hegyi *et al*., 2017 | No |
| Hildebrandt *et al*. 2017 | No |
| Hurkmans *et al*., 2020 | No |
| Koster *et al*., 2018 | Yes |
| Meijer *et al.* 2021 | Yes |
| Ruiz-Pinto *et al.*, 2017 | No |
| Sapkota *et al. 2021* | Yes |
| Sapkota *et al. 2022* | Yes |
| Visscher *et al.*, 2012 | Yes |
| Visscher *et al*., 2015 | Yes |
| Wang *et al*., 2014 | Yes |
| Wang *et al*., 2016 | Yes |
| Windsor *et al*., 2012 | No |
|  |  |
|  | **Treatment and outcome** |
| **Question** | **7. Were all patients treated according to label?** |
| Aminkeng *et al.*, 2015 | Yes |
| Bhuvaneshwar *et al.* 2019 | Yes |
| Caronia *et al*., 2011 | Yes |
| Chaix *et al.* 2020 | Yes |
| Gong *et al., 2021* | Yes |
| Hagleitner *et al*., 2015 | Yes |
| Hattinger *et al*. 2016 | Yes |
| Hegyi *et al*., 2017 | Yes |
| Hildebrandt *et al*. 2017 | Yes |
| Hurkmans *et al*., 2020 | Yes |
| Koster *et al*., 2018 | Yes |
| Meijer *et al.* 2021 | Yes |
| Ruiz-Pinto *et al.*, 2017 | Yes |
| Sapkota *et al. 2021* | Yes |
| Sapkota *et al. 2022* | Yes |
| Visscher *et al.*, 2012 | Yes |
| Visscher *et al*., 2015 | Yes |
| Wang *et al*., 2014 | Yes |
| Wang *et al*., 2016 | Yes |
| Windsor *et al*., 2012 | Yes |
|  |  |
| **Question** | **8. Were clinical data collected prospectively of retrospectively?** |
| Aminkeng *et al.*, 2015 | Retrospectively |
| Bhuvaneshwar *et al.* 2019 | Retrospectively |
| Caronia *et al*., 2011 | Retrospectively |
| Chaix *et al.* 2020 | Retrospectively |
| Gong *et al., 2021* | Retrospectively |
| Hagleitner *et al*., 2015 | Retrospectively |
| Hattinger *et al*. 2016 | Retrospectively |
| Hegyi *et al*., 2017 | Retrospectively |
| Hildebrandt *et al*. 2017 | Retrospectively |
| Hurkmans *et al*., 2020 | Retrospectively |
| Koster *et al*., 2018 | Retrospectively |
| Meijer *et al.* 2021 | Retrospectively |
| Ruiz-Pinto *et al.*, 2017 | Retrospectively |
| Sapkota *et al. 2021* | Retrospectively |
| Sapkota *et al. 2022* | Retrospectively and prospectively |
| Visscher *et al.*, 2012 | Retrospectively |
| Visscher *et al*., 2015 | Retrospectively |
| Wang *et al*., 2014 | Retrospectively |
| Wang *et al*., 2016 | Retrospectively |
| Windsor *et al*., 2012 | Retrospectively |
|  |  |
|  | **Follow-up and missing data** |
| **Question** | **9. Does the study describe the number of participants who withdrew and/or were lost to follow‐up after the start of the observation period?** |
| Aminkeng *et al.*, 2015 | No |
| Bhuvaneshwar *et al.* 2019 | No |
| Caronia *et al*., 2011 | No |
| Chaix *et al.* 2020 | No |
| Gong *et al., 2021* | No |
| Hagleitner *et al*., 2015 | No |
| Hattinger *et al*. 2016 | No |
| Hegyi *et al*., 2017 | No |
| Hildebrandt *et al*. 2017 | No |
| Hurkmans *et al*., 2020 | No |
| Koster *et al*., 2018 | No |
| Meijer *et al.* 2021 | No |
| Ruiz-Pinto *et al.*, 2017 | No |
| Sapkota *et al. 2021* | No |
| Sapkota *et al. 2022* | No |
| Visscher *et al.*, 2012 | No |
| Visscher *et al*., 2015 | No |
| Wang *et al*., 2014 | No |
| Wang *et al*., 2016 | No |
| Windsor *et al*., 2012 | Yes, three patients were excluded due to missing data |
|  |  |
| **Question** | **10. What was the overall percentage of participants who withdrew or were lost to follow‐up after the start of the observation period?** |
| Aminkeng *et al.*, 2015 | n/a |
| Bhuvaneshwar *et al.* 2019 | n/a |
| Caronia *et al*., 2011 | n/a |
| Chaix *et al.* 2020 | n/a |
| Gong *et al., 2021* | n/a |
| Hagleitner *et al*., 2015 | n/a |
| Hattinger *et al*. 2016 | n/a |
| Hegyi *et al*., 2017 | n/a |
| Hildebrandt *et al*. 2017 | n/a |
| Hurkmans *et al*., 2020 | n/a |
| Koster *et al*., 2018 | n/a |
| Meijer *et al.* 2021 | n/a |
| Ruiz-Pinto *et al.*, 2017 | n/a |
| Sapkota *et al. 2021* | n/a |
| Sapkota *et al. 2022* | n/a |
| Visscher *et al.*, 2012 | n/a |
| Visscher *et al*., 2015 | n/a |
| Wang *et al*., 2014 | n/a |
| Wang *et al*., 2016 | n/a |
| Windsor *et al*., 2012 | n/a |
|  |  |
| **Question** | **11. What was the overall percentage of missing data for the association between the pharmacogenetics interaction and the main outcome?** |
| Aminkeng *et al.*, 2015 | NR |
| Bhuvaneshwar *et al.* 2019 | NR |
| Caronia *et al*., 2011 | NR |
| Chaix *et al.* 2020 | NR |
| Gong *et al., 2021* | NR |
| Hagleitner *et al*., 2015 | NR |
| Hattinger *et al*. 2016 | NR |
| Hegyi *et al*., 2017 | NR |
| Hildebrandt *et al*. 2017 | NR |
| Hurkmans *et al*., 2020 | Yes, ranges from 8% to 66% (Table 2) |
| Koster *et al*., 2018 | NR |
| Meijer *et al.* 2021 | NR |
| Ruiz-Pinto *et al.*, 2017 | NR |
| Sapkota *et al. 2021* | NR |
| Sapkota *et al. 2022* | 21% for the discovery cohort |
| Visscher *et al.*, 2012 | NR |
| Visscher *et al*., 2015 | NR |
| Wang *et al*., 2014 | NR |
| Wang *et al*., 2016 | NR |
| Windsor *et al*., 2012 | NR |
|  |  |
|  | **Genotyping** |
| **Question** | **12. Specify genotyping method(s) used.** |
| Aminkeng *et al.*, 2015 | GWA array and allelic discrimination assays |
| Bhuvaneshwar *et al.* 2019 | Whole genome sequencing |
| Caronia *et al*., 2011 | Multiplex allelic discrimination assays |
| Chaix *et al.* 2020 | Exome sequencing |
| Gong *et al., 2021* | MassARRAY |
| Hagleitner *et al*., 2015 | ADME array |
| Hattinger *et al*. 2016 | Allelic discrimination assays |
| Hegyi *et al*., 2017 | Allelic discrimination assays |
| Hildebrandt *et al*. 2017 | Allelic discrimination assays |
| Hurkmans *et al*., 2020 | ADME array |
| Koster *et al*., 2018 | GWA array |
| Meijer *et al.* 2021 | GWA array |
| Ruiz-Pinto *et al.*, 2017 | GWA array |
| Sapkota *et al. 2021* | Whole genome sequencing |
| Sapkota *et al. 2022* | Whole genome sequencing |
| Visscher *et al.*, 2012 | ADME array |
| Visscher *et al*., 2015 | ADME array |
| Wang *et al*., 2014 | Cardiovascular SNP array |
| Wang *et al*., 2016 | GWA array |
| Windsor *et al*., 2012 | GWA array |
|  |  |
| **Question** | **13. For studies using genome‐wide association study (GWAS) data, specify the allele-calling algorithm used.** |
| Aminkeng *et al.*, 2015 | GenomeStudio |
| Bhuvaneshwar *et al.* 2019 | Sickle, Bowtie2, Samtools, Picard, and GATK’s HaplotypeCaller. |
| Caronia *et al*., 2011 | GenomeStudio |
| Chaix *et al.,* 2020 | Bwa mem aligner and Genome Analysis Toolkit version 3.8.0. |
| Gong *et al.*, 2021 | n/a |
| Hagleitner *et al*., 2015 | GenomeStudio |
| Hattinger *et al*. 2016 | n/a |
| Hegyi *et al*., 2017 | GenomeStudio |
| Hildebrandt *et al*. 2017 | n/a |
| Hurkmans *et al*., 2020 | DMET console software |
| Koster *et al*., 2018 | NR |
| Meijer *et al.* 2021 | NR |
| Ruiz-Pinto *et al.*, 2017 | GenomeStudio |
| Sapkota *et al. 2021* | Genome Analysis Toolkit’s HaplotypeCaller (GATK v3.4.0) |
| Sapkota *et al. 2022* | Burrows Wheeler Aligner (BWA-ALN v0.7.12) Genome Analysis Toolkit (GATK v3.4.0) |
| Visscher *et al.*, 2012 | NR |
| Visscher *et al*., 2015 | GenomeStudio |
| Wang *et al*., 2014 | NR |
| Wang *et al*., 2016 | NR |
| Windsor *et al*., 2012 | Beadstudio |
|  |  |
| **Question** | **14. How many SNPs were excluded based on call rate, and what cut-off point for exclusion was used?** |
| Aminkeng *et al.*, 2015 | Number of SNPs NR, cut-off was 0.95 |
| Bhuvaneshwar *et al.* 2019 | NR |
| Caronia *et al*., 2011 | 20 SNPs excluded in total (call rate+HWE), cut-off at 95% |
| Chaix *et al.* 2020 | NR |
| Gong *et al., 2021* | NR |
| Hagleitner *et al*., 2015 | 7 SNPs excluded with call rate <0.85 |
| Hattinger *et al*. 2016 | NR |
| Hegyi *et al*., 2017 | NR |
| Hildebrandt *et al*. 2017 | NR |
| Hurkmans *et al*., 2020 | 26 SNPs excluded with of call rate <0.9 |
| Koster *et al*., 2018 | Number of SNPs NR, cut-off was at 0.9 |
| Meijer *et al.* 2021 | Number of SNPs NR, cut-off was at 0.975 |
| Ruiz-Pinto *et al.*, 2017 | Number of SNPs NR, cut-off was at 0.99 |
| Sapkota *et al. 2021* | 84.3 million, cut-off at > 10% missingness |
| Sapkota *et al. 2022* | Number of SNPs NR, cut-off was at 0.90 |
| Visscher *et al.*, 2012 | NR |
| Visscher *et al*., 2015 | 374 SNPs excluded in QC, possibly due to low call rate, cut-off was at 0.95 |
| Wang *et al*., 2014 | 998 SNPs excluded with call rate < 0.95 |
| Wang *et al*., 2016 | 2999 SNPs excluded with call rate < 0.95 |
| Windsor *et al*., 2012 | NR |
|  |  |
| **Question** | **15. Was duplicate genotyping performed?** |
| Aminkeng *et al.*, 2015 | Yes |
| Bhuvaneshwar *et al.* 2019 | Yes |
| Caronia *et al*., 2011 | Yes, duplicate samples and CEPH trio's |
| Chaix *et al.* 2020 | NR |
| Gong *et al., 2021* | NR |
| Hagleitner *et al*., 2015 | NR |
| Hattinger *et al*. 2016 | NR |
| Hegyi *et al*., 2017 | NR |
| Hildebrandt *et al*. 2017 | Yes |
| Hurkmans *et al*., 2020 | No |
| Koster *et al*., 2018 | NR |
| Meijer *et al.* 2021 | NR |
| Ruiz-Pinto *et al.*, 2017 | Yes, 6 duplicate samples |
| Sapkota *et al. 2021* | NR |
| Sapkota *et al. 2022* | NR |
| Visscher *et al.*, 2012 | NR |
| Visscher *et al*., 2015 | Yes, 54 duplicate SNPs |
| Wang *et al*., 2014 | No |
| Wang *et al*., 2016 | No |
| Windsor *et al*., 2012 | NR |
|  |  |
| **Question** | **16. If duplicate genotyping was performed (Yes to #16), what was the duplicate genotyping concordance?** |
| Aminkeng *et al.*, 2015 | 100% |
| Bhuvaneshwar *et al.* 2019 | 9/20 (45%) in group A, 100% in group B and C |
| Caronia *et al*., 2011 | NR |
| Chaix *et al.* 2020 | n/a |
| Gong *et al., 2021* | n/a |
| Hagleitner *et al*., 2015 | n/a |
| Hattinger *et al*. 2016 | n/a |
| Hegyi *et al*., 2017 | n/a |
| Hildebrandt *et al*. 2017 | NR |
| Hurkmans *et al*., 2020 | n/a |
| Koster *et al*., 2018 | n/a |
| Meijer *et al.* 2021 | n/a |
| Ruiz-Pinto *et al.*, 2017 | NR |
| Sapkota *et al. 2021* | n/a |
| Sapkota *et al. 2022* | n/a |
| Visscher *et al.*, 2012 | n/a |
| Visscher *et al*., 2015 | Yes, 99.9% concordance |
| Wang *et al*., 2014 | n/a |
| Wang *et al*., 2016 | n/a |
| Windsor *et al*., 2012 | n/a |
|  |  |
| **Question** | **17. Was Hardy-Weinberg equilibrium calculated?** |
| Aminkeng *et al.*, 2015 | Yes |
| Bhuvaneshwar *et al.* 2019 | No |
| Caronia *et al*., 2011 | Yes |
| Chaix *et al.* 2020 | Yes |
| Gong *et al., 2021* | Yes |
| Hagleitner *et al*., 2015 | Yes |
| Hattinger *et al*. 2016 | Yes |
| Hegyi *et al*., 2017 | Yes |
| Hildebrandt *et al*. 2017 | NR |
| Hurkmans *et al*., 2020 | Yes |
| Koster *et al*., 2018 | Yes |
| Meijer *et al.* 2021 | Yes |
| Ruiz-Pinto *et al.*, 2017 | Yes |
| Sapkota *et al. 2021* | Yes |
| Sapkota *et al. 2022* | Yes |
| Visscher *et al.*, 2012 | Yes |
| Visscher *et al*., 2015 | Yes |
| Wang *et al*., 2014 | Yes |
| Wang *et al*., 2016 | Yes |
| Windsor *et al*., 2012 | Yes |
|  |  |
| **Question** | **18. If alleles were not in HWE, how were they handled?** |
| Aminkeng *et al.*, 2015 | Excluded if HWE P<1.0 × 10−4 |
| Bhuvaneshwar *et al.* 2019 | n/a |
| Caronia *et al*., 2011 | Excluded if deviated from HWE (threshold NR) |
| Chaix *et al.* 2020 | Excluded if HWE p<10^-6 |
| Gong *et al., 2021* | Included in the study |
| Hagleitner *et al*., 2015 | 31 variants with HWE p<0.05 were excluded |
| Hattinger *et al*. 2016 | Excluded if HWE p<0.01 |
| Hegyi *et al*., 2017 | Excluded if HWE p<0.05 |
| Hildebrandt *et al*. 2017 | n/a |
| Hurkmans *et al*., 2020 | 1 variant with HWE p<0.0001 was excluded |
| Koster *et al*., 2018 | Excluded if HWE p<10^-7 |
| Meijer *et al.* 2021 | Excluded if HWE p<10^-7 |
| Ruiz-Pinto *et al.*, 2017 | Excluded if HWE p<10^-8 |
| Sapkota *et al. 2021* | Excluded if HWE P<1.0 × 10-10 |
| Sapkota *et al. 2022* | Excluded if HWE P<1.0 × 10-10 |
| Visscher *et al.*, 2012 | 23 SNPs with HWE p<1.5*10-4 were excluded |
| Visscher *et al*., 2015 | 29 SNPs had HWE p<1.7 × 10-5. These SNPs were marked, but retained in the analysis. |
| Wang *et al*., 2014 | 108 SNPs with HWE p<0.000001 were excluded |
| Wang *et al*., 2016 | 3295 SNPs with HWE p<0.0001 were excluded |
| Windsor *et al*., 2012 | Excluded if HWE p<0.001 |
|  |  |
|  | **Statistical analyses** |
| **Question** | **19. How were missing data handled (select all that apply)?** |
| Aminkeng *et al.*, 2015 | NR |
| Bhuvaneshwar *et al.* 2019 | NR |
| Caronia *et al*., 2011 | NR |
| Chaix *et al.* 2020 | NR |
| Gong *et al., 2021* | NR |
| Hagleitner *et al*., 2015 | NR |
| Hattinger *et al*. 2016 | NR |
| Hegyi *et al*., 2017 | NR |
| Hildebrandt *et al*. 2017 | NR |
| Hurkmans *et al*., 2020 | Correction for number of datapoints per patient |
| Koster *et al*., 2018 | NR |
| Meijer *et al.* 2021 | NR |
| Ruiz-Pinto *et al.*, 2017 | NR |
| Sapkota *et al. 2021* | NR |
| Sapkota *et al. 2022* | Samples were excluded |
| Visscher *et al.*, 2012 | NR |
| Visscher *et al*., 2015 | NR |
| Wang *et al*., 2014 | NR |
| Wang *et al*., 2016 | NR |
| Windsor *et al*., 2012 | NR |
|  |  |
| **Question** | **20. What type of analysis was used for the main comparison?** |
| Aminkeng *et al.*, 2015 | NR |
| Bhuvaneshwar *et al.* 2019 | NR |
| Caronia *et al*., 2011 | NR |
| Chaix *et al.* 2020 | NR |
| Gong *et al., 2021* | NR |
| Hagleitner *et al*., 2015 | NR |
| Hattinger *et al*. 2016 | NR |
| Hegyi *et al*., 2017 | NR |
| Hildebrandt *et al*. 2017 | NR |
| Hurkmans *et al*., 2020 | NR |
| Koster *et al*., 2018 | NR |
| Meijer *et al.* 2021 | NR |
| Ruiz-Pinto *et al.*, 2017 | NR |
| Sapkota *et al. 2021* | NR |
| Sapkota *et al. 2022* | NR |
| Visscher *et al.*, 2012 | NR |
| Visscher *et al*., 2015 | NR |
| Wang *et al*., 2014 | NR |
| Wang *et al*., 2016 | NR |
| Windsor *et al*., 2012 | NR |
|  |  |
| **Question** | **21. For studies investigating more than one genetic variant, was adjustment for multiple testing applied?** |
| Aminkeng *et al.*, 2015 | Yes stage 1: threshold of P < 1 × 10−5, stage 2: threshold of p < 0.006, stage 3 threshold p < 0.05. |
| Bhuvaneshwar *et al.* 2019 | Yes, Benjamini Hochberg false discovery rate |
| Caronia *et al*., 2011 | Yes, corrected for 696 tests |
| Chaix *et al.* 2020 | Yes, Bonferroni |
| Gong *et al., 2021* | No |
| Hagleitner *et al*., 2015 | No |
| Hattinger *et al*. 2016 | No |
| Hegyi *et al*., 2017 | No |
| Hildebrandt *et al*. 2017 | No |
| Hurkmans *et al*., 2020 | Yes, Bonferroni |
| Koster *et al*., 2018 | Adjusted p-value threshold: SNPs with p<10^-4 were replicated in second patient cohort |
| Meijer *et al.* 2021 | Adjusted p-value threshold: SNPs with p<10^-5 were replicated in second patient cohort |
| Ruiz-Pinto *et al.*, 2017 | Yes, FDR correction |
| Sapkota *et al. 2021* | Yes, for variants with an minor allele frequency ≥ 0.05 the genome wide level of p<5x10-8 and for rare/low frequency variants (minor allele frequency <0.05) p < 7.3x10-8 |
| Sapkota *et al. 2022* | Yes, p<5x10-8 |
| Visscher *et al.*, 2012 | Yes, simpleM correction |
| Visscher *et al*., 2015 | Yes, a tiered analysis to identify SNPs associated at p < 0.01 in the larger discovery cohort that remained associated in the smaller replication cohort at p < 0.05. For combined cohort: Bonferroni corrected significance threshold at p<1.7*10^-5. |
| Wang *et al*., 2014 | Yes, according to Purcell et al. (P < 5 *10^-6) |
| Wang *et al*., 2016 | Yes, repeated sliding-window procedure |
| Windsor *et al*., 2012 | No |
|  |  |
| **Question** | **22. Were analyses performed to assess the effect of covariates?** |
| Aminkeng *et al.*, 2015 | Yes |
| Bhuvaneshwar *et al.* 2019 | NR |
| Caronia *et al*., 2011 | Yes |
| Chaix *et al.* 2020 | Yes |
| Gong *et al., 2021* | NR |
| Hagleitner *et al*., 2015 | Yes |
| Hattinger *et al*. 2016 | No |
| Hegyi *et al*., 2017 | Yes |
| Hildebrandt *et al*. 2017 | Yes |
| Hurkmans *et al*., 2020 | Yes |
| Koster *et al*., 2018 | Yes |
| Meijer *et al.* 2021 | Yes |
| Ruiz-Pinto *et al.*, 2017 | Yes |
| Sapkota *et al. 2021* | Yes |
| Sapkota *et al. 2022* | Yes |
| Visscher *et al.*, 2012 | Yes |
| Visscher *et al*., 2015 | NR |
| Wang *et al*., 2014 | Yes |
| Wang *et al*., 2016 | Yes |
| Windsor *et al*., 2012 | Yes |
|  |  |
|  | **Other** |
| **Question** | **23. Please provide any additional remarks (either positive or negative) on quality.** |
| Aminkeng *et al.*, 2015 | - |
| Bhuvaneshwar *et al.* 2019 | The results in the paper of the targeted DMET analysis do not correspond to the results in the supplementary material |
| Caronia *et al*., 2011 | - |
| Chaix *et al.* 2020 |  |
| Gong *et al., 2021* | - |
| Hagleitner *et al*., 2015 | - |
| Hattinger *et al*. 2016 | - |
| Hegyi *et al*., 2017 | - |
| Hildebrandt *et al*. 2017 | - |
| Hurkmans *et al*., 2020 | - |
| Koster *et al*., 2018 | - |
| Meijer *et al.* 2021 | - |
| Ruiz-Pinto *et al.*, 2017 | - |
| Sapkota *et al. 2021* | - |
| Sapkota *et al. 2022* |  |
| Visscher *et al.*, 2012 | - |
| Visscher *et al*., 2015 | - |
| Wang *et al*., 2014 | - |
| Wang *et al*., 2016 | - |
| Windsor *et al*., 2012 | - |

NR, not reported; n/a, not applicable

Table S7. Results question 5 of the quality assessment, regarding to reporting of relevant baseline characteristics. Characteristics were considered relevant if they were reported in 2 or more studies.

|  | Cardiotoxicity | | | | | | | | | | | Efficacy | | | | | | Other toxicities | | | | | |
| --- | --- | --- | --- | --- | --- | --- | --- | --- | --- | --- | --- | --- | --- | --- | --- | --- | --- | --- | --- | --- | --- | --- | --- |
| Outcome | **Aminkeng** *et al.*, 2015 | **Chaix** *et al.*, 2020 | **Hildebrandt** *et al*. 2017 | **Ruiz-Pinto** *et al.*, 2017 | **Sapkota** *et al.*, 2021 | **Sapkota** *et al.*, 2022 | **Visscher** *et al.*, 2012 | **Visscher** *et al*., 2015 | **Wang** *et al*., 2014 | **Wang** *et al*., 2016 | **Windsor** *et al*., 2012 | **Bhuvaneshwar** *et al.* 2019 | **Caronia** *et al*., 2011 | **Hagleitner** *et al*., 2015 | **Hattinger** *et al*. 2016 | **Koster** *et al*., 2018 | **Windsor** *et al*., 2012 | **Gong** *et al.*, 2021 | **Hattinger** *et al*. 2016 | **Hegyi** *et al*., 2017 | **Hurkmans** *et al*., 2020 | **Meijer** *et al.*, 2021 | **Windsor** *et al*., 2012 |
| Age | x | x | x | x | x | x | x |  | x | x | x |  | x | x | x | x | x | x | x | x | x | x | x |
| Year of diagnosis |  |  |  |  |  |  |  |  |  |  |  |  |  |  |  |  |  |  |  |  |  | x |  |
| Sex | x | x | x | x | x | x | x |  | x | x | x |  | x | x | x | x | x | X | x | x | x | X | x |
| Primary tumor diagnosis | x | x | x | x |  |  | x |  | x | x | x |  | x | x | x | x | x | X | x | x | x | X | x |
| Age at follow-up |  |  |  |  | x | x |  |  |  |  |  |  |  |  |  |  |  |  |  |  |  | x |  |
| Age at cardiac dysfunction |  |  |  |  |  | x |  |  |  |  |  |  |  |  |  |  |  |  |  |  |  |  |  |
| Follow-up time | x |  | x | x |  |  | x |  | x | x | x |  |  |  |  |  | x |  |  |  |  | X | x |
| Metastasis at diagnosis |  |  |  |  |  |  |  |  |  |  | x |  | x | x | x | x | x |  | x |  | x |  | x |
| Radiation involving heart | x | x | x | x | x | x | x |  | x | x |  |  |  |  |  |  |  |  |  |  |  |  |  |
| Ethnicity |  |  | x |  |  |  |  |  | x | x | x |  |  |  |  |  | x |  |  |  | x |  | x |
| Anthracycline cumulative dose | x | x | x | x | x | x | x |  | x |  |  |  |  |  |  |  |  |  |  |  |  |  |  |
| Cisplatin cumulative dose |  |  |  |  |  |  |  |  |  |  |  |  |  |  |  |  |  |  |  |  |  | x |  |
| Additional carboplatin |  |  |  |  |  |  |  |  |  |  |  |  |  |  |  |  |  |  |  |  |  | x |  |
| Treatment duration |  | x |  |  |  |  |  |  |  |  |  |  |  |  |  |  |  |  |  |  |  |  |  |
| Time from first dose to last follow-up or lowest ejection fraction |  | x |  |  |  | x |  |  |  |  |  |  |  |  |  |  |  |  |  |  |  |  |  |
| Tumor in axial skeleton/ bone tumor location |  |  |  |  |  |  |  |  |  |  | x |  | x | x | x |  | x |  | x |  |  |  | x |
| Histological subtype |  |  |  |  |  |  |  |  |  |  | x |  |  |  | x |  | x |  | x |  |  |  | x |
| Survival state |  |  |  |  |  |  |  |  |  |  | x |  | x |  |  | x | x |  |  |  |  |  | x |
| Anthracycline type | x |  |  | x |  |  | x |  |  |  |  |  |  |  |  |  |  |  |  |  |  |  |  |
| Dexrazoxane use |  | x |  |  |  |  |  |  |  |  |  |  |  |  |  |  |  |  |  |  |  |  |  |
| Ejection fraction |  | x | x |  | x | x |  |  | x | x |  |  |  |  |  |  |  |  |  |  |  |  |  |
| Risk group |  |  | x |  |  |  |  |  |  |  |  |  |  |  |  |  |  |  |  | x |  |  |  |
| Relapse |  |  |  |  |  |  |  |  |  |  | x |  | x |  |  |  | x |  |  |  |  |  | x |
| Weight |  |  |  |  |  |  |  |  |  |  |  |  |  |  |  |  |  | x |  |  |  |  |  |
| MTX Dose |  |  |  |  |  |  |  |  |  |  |  |  |  |  |  |  |  | x |  |  |  |  |  |
| MTX plasma levels |  |  |  |  |  |  |  |  |  |  |  |  | x |  |  |  |  |  |  |  | x |  |  |
| # characteristics reported in this article | **7** | **9** | **9** | **7** | **6** | **8** | **7** | **0** | **8** | **7** | **10** | **0** | **8** | **5** | **6** | **5** | **10** | **5** | **6** | **4** | **6** | **8** | **10** |

REFERENCES

1. Clemens, E., et al., *Genetic variation of cisplatin-induced ototoxicity in non-cranial-irradiated pediatric patients using a candidate gene approach: The International PanCareLIFE Study.* Pharmacogenomics J, 2020. **20**(2): p. 294-305.

2. Feng, W., et al., *The effects of common variants in MDM2 and GNRH2 genes on the risk and survival of osteosarcoma in Han populations from Northwest China.* Sci Rep, 2020. **10**(1): p. 15939.

3. Goričar, K., et al., *Influence of the folate pathway and transporter polymorphisms on methotrexate treatment outcome in osteosarcoma.* Pharmacogenet Genomics, 2014. **24**(10): p. 514-21.

4. Goričar, K., et al., *Genetic variability of DNA repair mechanisms and glutathione-S-transferase genes influences treatment outcome in osteosarcoma.* Cancer Epidemiol, 2015. **39**(2): p. 182-8.

5. Kang, C., et al., *Relationship of common variants in Interleukin 33 gene with susceptibility and prognosis of osteosarcoma in Han Chinese population.* J Cancer, 2019. **10**(5): p. 1138-1144.

6. Langer, T., et al., *Usefulness of current candidate genetic markers to identify childhood cancer patients at risk for platinum-induced ototoxicity: Results of the European PanCareLIFE cohort study.* Eur J Cancer, 2020. **138**: p. 212-224.

7. Lanvers-Kaminsky, C., et al., *Human OCT2 variant c.808G>T confers protection effect against cisplatin-induced ototoxicity.* Pharmacogenomics, 2015. **16**(4): p. 323-32.

8. Lui, G., et al., *A Pharmacokinetic and Pharmacogenetic Analysis of Osteosarcoma Patients Treated With High-Dose Methotrexate: Data From the OS2006/Sarcoma-09 Trial.* J Clin Pharmacol, 2018. **58**(12): p. 1541-1549.

9. Ruiz-Pinto, S., et al., *Exome array analysis identifies ETFB as a novel susceptibility gene for anthracycline-induced cardiotoxicity in cancer patients.* Breast Cancer Res Treat, 2018. **167**(1): p. 249-256.

10. Sági, J.C., et al., *Possible roles of genetic variations in chemotherapy related cardiotoxicity in pediatric acute lymphoblastic leukemia and osteosarcoma.* BMC Cancer, 2018. **18**(1): p. 704.

11. Spracklen, T.F., et al., *Genetic variation in Otos is associated with cisplatin-induced ototoxicity.* Pharmacogenomics, 2014. **15**(13): p. 1667-76.

12. Sun, Y., et al., *Genetic polymorphisms in nucleotide excision repair pathway influences response to chemotherapy and overall survival in osteosarcoma.* Int J Clin Exp Pathol, 2015. **8**(7): p. 7905-12.

13. Vargas-Neri, J.L., et al., *Pharmacogenomic study of anthracycline-induced cardiotoxicity in Mexican pediatric patients.* Pharmacogenomics, 2022. **23**(5): p. 291-301.

14. Visscher, H., et al., *Validation of variants in SLC28A3 and UGT1A6 as genetic markers predictive of anthracycline-induced cardiotoxicity in children.* Pediatr Blood Cancer, 2013. **60**(8): p. 1375-81.
