# Supplementary material for: Pharmacogenetics of chemotherapy treatment response and -toxicities in patients with osteosarcoma: a systematic review
Source: BMC Cancer. 2022 Dec 19;22:1326. doi: 10.1186/s12885-022-10434-5 (PMC9761983; doi:10.1186/s12885-022-10434-5)
Supplement: Supplementary file 2 — Additional file 2: Table S8. Characteristics and results of independent discovery and replication cohorts, studying genetic variants associated with treatment response in patients with osteosarcoma. Table S9. Characteristics and results of independent discovery and replication cohorts, studying genetic variants associated with doxorubicin-induced cardiotoxicity. Table S10. Characteristics and results of independent discovery and replication cohorts, studying genetic variants associated with bone marrow- hepato- and nephrotoxicity after treatment with cisplatin, doxorubicin or methotrexate. [file 12885_2022_10434_MOESM2_ESM.pdf]

### Replication studies

Table S8. Characteristics and results of independent discovery and replication cohorts, studying genetic variants associated with treatment response in patients with osteosarcoma.

| Author | Outcome | No. of patients | HR (95% CI) | P value | Ref |
|---|---|---|---|---|---|
| ***ABCB1* rs10276036** | | | | | |
| Caronia *et al.,* 2011* | EFS | 102 | 0.42 (0.29-0.81) | **0.0021** | (1) |
| Liu *et al.*, 2014 | PFS | 186 | 1.24 (0.79-1.93) | 0.32 | (2) |
| ***ABCB1* rs1128503** | | | | | |
| Caronia *et al.,* 2011* | EFS | 102 | 0.42 (0.29-0.81) | **0.0021** | (1) |
| Windsor *et al.,* 2012 | PFS | 58 | N/S | ns | (3) |
| Yang *et al.*, 2013 | DFS | 208 | 3.74 (1.63-7.4) | **0.003** | (4) |
| Li *et al.*, 2014 | OS | 162 | 3.17 (1.14-6.67) | **0.01** | (5) |
| Liu *et al.*, 2014 | PFS | 186 | 0.49 (0.31-0.76) | **<0.001** | (2) |
| Hattinger et al., 2016 | EFS | 126 | N/S | ns | (6) |
| ***ABCB1* rs4148737** | | | | | |
| Caronia *et al.,* 2011* | EFS | 102 | 2.6 (1.24-3.22) | **0.00051** | (1) |
| Liu *et al.*, 2014 | PFS | 186 | 1.47 (0.93-2.33) | 0.08 | (2) |
| ***ABCC2* rs2273697** | | | | | |
| Hattinger *et al.,* 2016* | EFS | 126 | N/S | **0.049** | (6) |
| Windsor *et al.,* 2012 | PFS | 58 | N/S | ns | (3) |
| ***ABCC2* rs717620** | | | | | |
| Windsor *et al.,* 2012* | Histological response | 58 | 6.3 (1.4-28.5)[a] | **0.017** | (3) |
| Yang *et al.*, 2013 | DFS | 208 | N/S | ns | (4) |
| Goričar *et al.*, 2014 | OS | 44 | 0.5 (0.14-1.75) | 0.275 | (7) |
| Hagleitner *et al.,* 2015 | PFS | 126 | N/S | ns | (8) |
| Hattinger et al., 2016 | EFS | 126 | N/S | ns | (6) |
| ***ABCC3* rs4148416** | | | | | |
| Caronia *et al.,* 2011* | EFS | 102 | 6.33 (1.79-12.7) | **0.00028** | (1) |
| Yang *et al.*, 2013 | DFS | 208 | 4.32 (1.75-15.65) | **0.006** | (4) |
| Liu *et al.*, 2014 | PFS | 186 | 2.73 (1.62-4.66) | **<0.001** | (2) |
| ***ABCC5* rs939338** | | | | | |
| Hagleitner *et al.,* 2015* | PFS | 126 | 1.86 (1.06-3.24)[a] | **0.03** | (8) |
| Hagleitner *et al.,* 2015 | PFS | 64 | 1.36 (0.62-2.98)[a] | 0.44 | (8) |
| Xu *et al.*, 2018 | PFS | 132 | 2.01 (1.2-3.37)[a] | **0.008** | (9) |
| ***CASP3* rs2720376** | | | | | |

| | | | | | |
|---|---|---|---|---|---|
| Hagleitner *et al.,* 2015* | PFS | 126 | 0.52 (0.3-0.9)[a] | **0.02** | (8) |
| Hagleitner *et al.,* 2015 | PFS | 64 | 0.68 (0.3-1.56)[a] | 0.36 | (8) |
| Xu *et al.*, 2018 | PFS | 132 | 1.02 (0.62-1.71)[a] | 0.95 | (9) |
| ***CCND1* rs9344** | | | | | |
| Windsor *et al.,* 2012* | PFS | 58 | N/S | **0.018** | (3) |
| ***CHST12* rs3735099** | | | | | |
| Bhuvaneshwar *et al.,* 2019* | OS and tumor necrosis | 100 | 3.124 (N/S) | **0.04** | (10) |
| ***CHST12* rs3735100** | | | | | |
| Bhuvaneshwar *et al.,* 2019* | OS and tumor necrosis | 100 | 3.124 (N/S) | **0.04** | (10) |
| ***CYP2B6*6*** | | | | | |
| Hattinger *et al.,* 2016* | EFS | 126 | N/S | **0.039** | (6) |
| ***CYP3A4* rs4646437** | | | | | |
| Hagleitner *et al.,* 2015* | PFS | 126 | 0.34 (0.13-0.85)[a] | **0.02** | (8) |
| Hagleitner *et al.,* 2015 | PFS | 64 | 0.61 (0.19-1.94)[a] | 0.4 | (8) |
| Xu *et al.*, 2018 | PFS | 132 | 0.69 (0.39-1.26)[a] | 0.24 | (9) |
| ***FasL* rs763110** | | | | | |
| Hagleitner *et al.,* 2015* | PFS | 126 | 1.97 (1.04-3.73)[a] | **0.04** | (8) |
| Hagleitner *et al.,* 2015 | PFS | 64 | 1.5 (0.7-3.21)[a] | 0.3 | (8) |
| Xu *et al.*, 2018 | PFS | 132 | 2.26 (1.35-3.77)[a] | **0.002** | (9) |
| ***GGH* rs11545078** | | | | | |
| Hattinger *et al.,* 2016* | EFS | 126 | N/S | **0.037** | (6) |
| ***GLDC* rs3765555** | | | | | |
| Koster *et al.,* 2018* | OS | 523 | 1.71 (1.34-2.18) | $\mathbf{1.60\times10^{-5}}$ | (11) |
| Koster *et al.,* 2018 | OS | 109 | 2.12 (1.27-3.53) | $\mathbf{3.87\times10^{-3}}$ | (11) |
| ***GLDC* rs55933544** | | | | | |
| Koster *et al.,* 2018* | OS | 523 | 1.91 (1.49-2.45) | $\mathbf{3.20\times10^{-7}}$ | (11) |
| Koster *et al.,* 2018 | OS | 109 | 1.98 (1.16-3.38) | **0.012** | (11) |
| Lin *et al.*, 2020 | OS | 72 | 3.98 (1.87-4.96)[a] | **<0.001** | (12) |
| ***GSTP1* rs1695** | | | | | |
| Windsor *et al.,* 2012* | Histological response | 58 | 7.9 (1.5-42.5)[a] | **0.016** | (3) |
| Windsor *et al.,* 2012* | PFS | 58 | N/S | **0.025** | (3) |
| Yang *et al.*, 2012 | OS | 187 | 0.53 (0.24-1.16) | 0.32 | (13) |
| Zhang *et al.*, 2012 | EFS | 159 | 2.35 (1.13-4.85) | N/S | (14) |
| Teng *et al.*, 2013 | OS | 146 | 2.73 (1.05-7.45) | **<0.05** | (15) |
| Li *et al.*, 2014 | OS | 162 | 3.86 (1.41-10.2) | **0.004** | (5) |

| | | | | | |
|---|---|---|---|---|---|
| Liu *et al.*, 2014 | PFS | 186 | 2.16 (1.38-3.38) | **<0.001** | (2) |
| Hattinger et al., 2016 | EFS | 126 | N/S | ns | (6) |
| ***GSTT1* null** | | | | | |
| Windsor *et al.,* 2012* | PFS | 58 | N/S | **0.006** | (3) |
| Yang *et al.*, 2012 | OS | 187 | 0.92 (0.57-1.76) | 0.75 | (13) |
| Zhang *et al.*, 2012 | EFS | 159 | 0.7 (0.39-1.33) | ns | (14) |
| Teng *et al.*, 2013 | OS | 146 | 1.43 (0.72-3.04) | 0.31 | (15) |
| Li *et al.*, 2014 | OS | 162 | 1.26 (0.63-2.42) | 0.66 | (5) |
| Liu *et al.*, 2014 | PFS | 186 | 1.18 (0.64-2.18) | 0.58 | (2) |
| Hattinger et al., 2016 | EFS | 126 | N/S | ns | (6) |
| ***MSH2* rs4638843** | | | | | |
| Hagleitner *et al.,* 2015* | PFS | 126 | 2.32 (1.02-5.27)[a] | **0.04** | (8) |
| Hagleitner *et al.,* 2015 | PFS | 64 | 3.86 (0.98-15.25)[a] | **0.05** | (8) |
| ***MTHFD1* rs2236225** | | | | | |
| Windsor *et al.,* 2012* | Histological response | 58 | 0.2 (0.05-0.9)[a] | **0.03** | (3) |
| Goričar *et al.*, 2014 | OS | 44 | 1.52 (0.54-4.27) | 0.43 | (7) |
| Hattinger et al., 2016 | EFS | 126 | N/S | ns | (6) |
| ***RFC1/SLC19A1* rs1051266** | | | | | |
| Windsor *et al.,* 2012* | PFS | 58 | N/S | **0.02** | (3) |
| Goričar *et al.*, 2014 | OS | 44 | 0.77 (0.29-2.07) | 0.601 | (7) |
| Jabeen *et al.*, 2015 | OS | 62 | N/S | **0.046** | (16) |
| Hattinger et al., 2016 | EFS | 126 | N/S | ns | (6) |
| ***SLC22A1* rs4646272** | | | | | |
| Bhuvaneshwar *et al.,* 2019* | OS and tumor necrosis | 100 | 3.723 (N/S) | **0.007** | (10) |
| ***SLC22A8* rs2187384** | | | | | |
| Bhuvaneshwar *et al.,* 2019* | OS and tumor necrosis | 100 | 3.161 (N/S) | **0.019** | (10) |
| ***TP53* rs1642785** | | | | | |
| Hattinger *et al.,* 2016* | EFS | 126 | N/S | **0.01** | (6) |
| ***UGT2B15* rs34073924** | | | | | |
| Bhuvaneshwar *et al.,* 2019* | OS and tumor necrosis | 100 | 3.462 (N/S) | **0.024** | (10) |

N/S, not specified; ns, not significant; HR, hazard ratio; OS, overall survival; PFS, progression-free survival; EFS, event-free survival; DFS, disease-free survival

* discovery study that was identified in systematic literature search

[a] Odds ratio (95% CI)

Table S9. Characteristics and results of independent discovery and replication cohorts, studying genetic variants associated with doxorubicin-induced cardiotoxicity.

| | Outcome | Patient cohort | Diagnosis | No. of patients | OR (95% CI) | P value | Ref. |
|---|---|---|---|---|---|---|---|
| ***ATP2B1* rs17249754** | | | | | | | |
| Hildebrandt *et al.,* 2017* | EF 45–50% and symptoms; or FS ≤ 25% and/or EF ≤ 45% | Pediatric | Mixed cancer cohort | 108 | 0.33 (0.12–0.92) | **0.034** | (17) |
| ***CELF4* rs1786814** | | | | | | | |
| Wang *et al*., 2016* | FS ≤ 28% or LVEF ≤ 40% or symptoms | Pediatric | Mixed cancer cohort | 331 | 10.16 (3.8-27.3) | **<0.001** | (18) |
| Wang *et al*., 2016 | FS ≤ 28% or LVEF ≤ 40% or symptoms | Pediatric | Mixed cancer cohort | 75 | 5.09 (1.03-25.23) | **0.046** | (18) |
| Leger *et al*., 2016 | ICD codes of symptoms or self-reported through surveys | Adult | Hematopoietic cell transplantation survivors | 576 | 22.2 (1.5-339.2) | **0.01** | (19) |
| ***GPR35* rs12468485** | | | | | | | |
| Ruiz-Pinto *et al*., 2017* | FS ≤ 27% | Pediatric | Mixed cancer cohort | 93 | N/A | **$7\times10^{-6}$** | (20) |
| ***GSTP1* rs1695** | | | | | | | |
| Windsor *et al.,* 2012* | EF decrease with ≥1 CTCAE | Pediatric | Osteosarcoma | 58 | 4.8 (1.4-16.4) | 0.011 | (3) |
| ***HAS3* rs2232228** | | | | | | | |
| Wang *et al*., 2014* | FS ≤ 28% or LVEF ≤ 40% or symptoms | Pediatric | Mixed cancer cohort | 401 | 8.9 (2.1-37.5) | **0.003** | (21) |
| Wang *et al*., 2014 | FS ≤ 28% or LVEF ≤ 40% or symptoms | Pediatric | Mixed cancer cohort | 76 | 4.5 (1.1-18.7) | **0.04** | (21) |
| Wang *et al*., 2016 | FS ≤ 28% or LVEF ≤ 40% or symptoms | Pediatric | Mixed cancer cohort | 331 | N/S | 0.6 | (18) |
| Leger *et al*., 2016 | ICD codes of symptoms or self-reported through surveys | Adult | Hematopoietic cell transplantation survivors | 576 | 21.8 (1.2-386.4) | **0.02** | (19) |
| Ságí *et al*., 2018 | FS ≤ 28% | Pediatric | ALL and osteosarcoma | 661 | N/S | N/S, ns | (22) |
| ***PLCE1* rs932764** | | | | | | | |
| Hildebrandt *et al.,* 2017* | EF 45–50% and symptoms; or FS ≤ 25% and/or EF ≤ 45% | Pediatric | Mixed cancer cohort | 108 | 0.48 (0.27–0.85) | **0.012** | (17) |
| ***RARG* rs2229774** | | | | | | | |
| Aminkeng *et al*., 2015* | FS ≤ 24% or symptoms requiring intervention in CTCAE v3 | Pediatric | Mixed cancer cohort | 280 | 7 (2.9-17) | **$5.0\times10^{-6}$** | (23) |
| Aminkeng *et al*., 2015 | FS ≤ 24% or symptoms requiring intervention in CTCAE v3 | Pediatric | Mixed cancer cohort | 80 | N/A | **$4.3\times10^{-11}$** | (23) |
| Aminkeng *et al*., 2015 | FS ≤ 24% or symptoms requiring intervention in CTCAE v3 | Pediatric | Mixed cancer cohort | 96 | 4.1 (1.5-11.5) | **0.0043** | (23) |
| Serie *et al*., 2017 | LVEF reduction > 10% or LVEF < 53% | Adult | HER2+ breast cancer | 1191 | 2.39 (0.91-6.23) | 0.076 | (24) |
| Schneider *et al*., 2017 | LVEF reduction > 20% or LVEF < 50% | Adult | Breast cancer | 102 | 0.11 (N/S) | **0.0000041** | (25) |
| Ságí *et al*., 2018 | FS ≤ 28% | Pediatric | ALL and osteosarcoma | 661 | N/S | N/S, ns | (22) |
| Park *et al*., 2020 | LVEF reduction > 10% or LVEF < 50% | Adult | Breast cancer | 257 | N/S | N/S, ns | (26) |

| | | | | | | | |
|---|---|---|---|---|---|---|---|
| ***SLC22A17* rs4982753** | | | | | | | |
| Visscher *et al.*, 2015* | FS ≤ 26% or symptoms requiring intervention in CTCAE v3 | Pediatric | Mixed cancer cohort | 335 | 0.52 (0.31-0.85) | **0.0078** | (27) |
| Visscher *et al.*, 2015 | FS ≤ 26% or symptoms requiring intervention in CTCAE v3 | Pediatric | Mixed cancer cohort | 185 | 0.39 (0.19-0.81) | **0.0071** | (27) |
| Schneider *et al.*, 2017 | LVEF reduction > 20% or LVEF < 50% | Adult | Breast cancer | 102 | N/S | N/S, ns | (25) |
| Sági *et al.*, 2018 | FS ≤ 28% | Pediatric | ALL and osteosarcoma | 661 | N/S | N/S, ns | (22) |
| ***SLC22A7* rs4149178** | | | | | | | |
| Visscher *et al.*, 2015* | FS ≤ 26% or symptoms requiring intervention in CTCAE v3 | Pediatric | Mixed cancer cohort | 335 | 0.41 (0.21-0.77) | **0.0034** | (27) |
| Visscher *et al.*, 2015 | FS ≤ 26% or symptoms requiring intervention in CTCAE v3 | Pediatric | Mixed cancer cohort | 185 | 0.39 (0.14-1.05) | **0.047** | (27) |
| Schneider *et al.*, 2017 | LVEF reduction > 20% or LVEF < 50% | Adult | Breast cancer | 102 | N/S | N/S, ns | (25) |
| Sági *et al.*, 2018 | FS ≤ 28% | Pediatric | ALL and osteosarcoma | 661 | N/S | N/S, ns | (22) |
| ***SLC28A3* rs7853758** | | | | | | | |
| Visscher *et al.*, 2012* | FS ≤ 26% or symptoms requiring intervention in CTCAE v3 | Pediatric | Mixed cancer cohort | 156 | 0.29 (0.11-0.81) | **0.0071** | (28) |
| Visscher *et al.*, 2012 | FS ≤ 26% or symptoms requiring intervention in CTCAE v3 | Pediatric | Mixed cancer cohort | 188 | 0.33 (0.13-0.8) | **0.0072** | (28) |
| Visscher *et al.*, 2012 | FS ≤ 26% or symptoms requiring intervention in CTCAE v3 | Pediatric | Mixed cancer cohort | 96 | 0.69 (N/S) | 0.38 | (28) |
| Visscher *et al.*, 2013 | FS ≤ 26% or symptoms requiring intervention in CTCAE v3 | Pediatric | Mixed cancer cohort | 177 | 0.46 (0.2-1.08) | 0.058 | (29) |
| Vulsteke *et al.*, 2015 | LVEF reduction > 10% or CTCAEv4 grade 3-5 | Adult | Breast cancer | 877 | N/S | N/S, ns | (30) |
| Reichwagen *et al.*, 2015 | CTCAE v2 > grade 0 | Adult | CD20+ B-cell lymphoma | 450 | 1.4 (0.8-2.3) | 0.27 | (31) |
| Reichwagen *et al.*, 2015 | CTCAE v2 > grade 0 | Adult | CD20+ B-cell lymphoma | 634 | 1.4 (0.7-3) | 0.39 | (31) |
| Hertz *et al.*, 2016 | EF < 55% | Adult | Breast cancer | 166 | 0.55 (0.16-1.91) | 0.43 | (32) |
| Ruiz-Pinto *et al.*, 2017 | FS ≤ 27% | Pediatric | Mixed cancer cohort | 93 | N/S | **<0.05** | (20) |
| Serie *et al.*, 2017 | LVEF reduction > 10% or LVEF < 53% | Adult | HER2+ breast cancer | 1191 | 0.71 (0.25-2.02) | 0.52 | (24) |
| Sági *et al.*, 2018 | FS ≤ 28% | Pediatric | ALL and osteosarcoma | 661 | 9.837 (1.73-56.02) | **0.01** | (22) |

N/S, not specified; N/A, not applicable; ns, not significant; FS, fractional shortening ;LVEF, left ventricular ejection fraction; EF, ejection fraction; CTCAE, Common Terminology Criteria for Adverse Events; ALL, acute lymphatic leukemia

* discovery study that was identified in systematic literature search

Table S10. Characteristics and results of independent discovery and replication cohorts, studying genetic variants associated with bone marrow- hepato- and nephrotoxicity after treatment with cisplatin, doxorubicin or methotrexate.

| **Author** | **Outcome** | **Induced by**[a] | **Patient cohort** | **Diagnosis** | **No. of patients** | **OR (95% CI)** | **p-value** | **Ref.** |
|---|---|---|---|---|---|---|---|---|
| ***ABCB1*** **rs1128503** | **Hepatotoxicity** | | | | | | | |
| Hattinger et al., 2016* | CTCAE v4 grade 4 | MAP | Pediatric | Osteosarcoma | 57 | 2.06 (1.16-3.66) | **0.014** | (6) |
| Hegyi et al., 2017 | CTCAE v3 grade 3-4 | MTX | Pediatric | Osteosarcoma | 59 | N/S | ns | (33) |
| Hurkmans et al., 2020 | ALAT | MTX | Pediatric | Osteosarcoma | 113 | N/S | ns | (34) |
| ***ABCC2*** **rs17222723** | **Leukopenia** | | | | | | | |
| Windsor et al., 2012* | CTCAE v3 grade 3-4 | MTX | Pediatric | Osteosarcoma | 58 | 5.2 (1.2-22.4) | **0.028** | (3) |
| Hurkmans et al., 2020 | Leukocyte counts | MTX | Pediatric | Osteosarcoma | 113 | N/S | ns | (34) |
| ***ABCC2*** **rs2273697** | **Hepatotoxicity** | | | | | | | |
| Windsor et al., 2012 | CTCAE v3 grade 3-4 | MAP | Pediatric | Osteosarcoma | 58 | N/S | ns | (3) |
| Sharifi et al., 2014 | CTCAE v3 grade 1-4 | MTX | Pediatric | ALL | 65 | 0.55 (0.2-1.48) | 0.32 | (35) |
| Goričar et al., 2014 | CTCAE v4 grade 3-4 | MTX | Pediatric | Osteosarcoma | 118 | 1.88 (0.1-3.49) | 0.414 | (7) |
| Hattinger et al., 2016* | CTCAE v4 grade 4 | MAP | Pediatric | Osteosarcoma | 57 | 1.96 (1.2-3.2) | **0.007** | (6) |
| Gervasini et al., 2017 | CTCAE v4 | MTX | Pediatric | ALL | 41 | N/S | ns | (36) |
| Hegyi et al., 2017 | CTCAE v3 grade 3-4 | MTX | Pediatric | Osteosarcoma | 59 | N/S | ns | (33) |
| Hurkmans et al., 2020 | ALAT | MTX | Pediatric | Osteosarcoma | 113 | N/S | ns | (34) |
| ***ABCC2*** **rs2273697** | **Leukopenia** | | | | | | | |
| Windsor et al., 2012 | CTCAE v3 grade 3-4 | MAP | Pediatric | Osteosarcoma | 58 | N/S | ns | (3) |
| Sharifi et al., 2014 | CTCAE v3 grade 1-4 | MTX | Pediatric | ALL | 65 | 0.61 (0.13-2.97) | 0.69 | (35) |
| Hattinger et al., 2016* | CTCAE v4 grade 4 | MAP | Pediatric | Osteosarcoma | 57 | 13.16 (1.56-111.12) | **0.018** | (6) |
| Gervasini et al., 2017 | CTCAE v4 | MTX | Pediatric | ALL | 41 | N/S | ns | (36) |
| Hegyi et al., 2017* | CTCAE v3 grade 3-4 | MTX | Pediatric | Osteosarcoma | 59 | 3.3 (1.2-9.4) | **0.02** | (33) |
| Hurkmans et al., 2020 | Leukocyte counts | MTX | Pediatric | Osteosarcoma | 113 | N/S | ns | (34) |
| ***ABCC2*** **rs2273697** | **Thrombocytopenia** | | | | | | | |
| Windsor et al., 2012 | CTCAE v3 grade 3-4 | MAP | Pediatric | Osteosarcoma | 58 | N/S | ns | (3) |
| Sharifi et al., 2014 | CTCAE v3 grade 1-4 | MTX | Pediatric | ALL | 65 | 2.5 (0.89-6.97) | 0.12 | (35) |
| Hattinger et al., 2016* | CTCAE v4 grade 4 | MAP | Pediatric | Osteosarcoma | 57 | 4.33 (1.2-15.63) | **0.025** | (6) |
| Gervasini et al., 2017 | CTCAE v4 | MTX | Pediatric | ALL | 41 | N/S | ns | (36) |
| Hurkmans et al., 2020 | Thrombocyte counts | MTX | Pediatric | Osteosarcoma | 113 | N/S | ns | (34) |
| ***ABCC2*** **rs3740066** | **Leukopenia** | | | | | | | |
| Hattinger et al., 2016 | CTCAE v4 grade 4 | MAP | Pediatric | Osteosarcoma | 57 | N/S | ns | (6) |
| Hegyi et al., 2017* | CTCAE v3 grade 3-4 | MTX | Pediatric | Osteosarcoma | 59 | 0.4 (0.2-0.9) | **0.02** | (33) |

| | | | | | | | | |
|---|---|---|---|---|---|---|---|---|
| Hurkmans et al., 2020 | Leukocyte counts | MTX | Pediatric | Osteosarcoma | 113 | N/S | ns | (34) |
| ***CYBA* rs4673** | **Anemia** | | | | | | | |
| Windsor et al., 2012* | CTCAE v3 grade 3-4 | Doxorubicin, cisplatin | Pediatric | Osteosarcoma | 58 | 0.3 (0.09-0.9) | **0.038** | (3) |
| ***CYP2B6* rs4803418** | **Thrombocytopenia** | | | | | | | |
| Hurkmans et al., 2020* | Thrombocyte counts | MTX | Pediatric | Osteosarcoma | 113 | -0.19 (-0.27--0.1) [b] | **0.00003** | (34) |
| ***CYP2B6* rs4803419** | **Thrombocytopenia** | | | | | | | |
| Hurkmans et al., 2020* | Thrombocyte counts | MTX | Pediatric | Osteosarcoma | 113 | 0.19 (0.1-0.29) [b] | **0.00006** | (34) |
| ***CYP4F8* rs4808326** | **Thrombocytopenia** | | | | | | | |
| Hurkmans et al., 2020* | Thrombocyte counts | MTX | Pediatric | Osteosarcoma | 113 | -0.19 (-0.28--0.09) [b] | **0.00009** | (34) |
| ***ERCC1* rs3212986** | **Leukopenia** | | | | | | | |
| Windsor et al., 2012* | CTCAE v3 grade 3-4 | Doxorubicin | Pediatric | Osteosarcoma | 58 | 5.4 (1.1-26) | **0.036** | (3) |
| Hattinger et al., 2016 | CTCAE v4 grade 4 | MAP | Pediatric | Osteosarcoma | 57 | N/S | ns | (6) |
| ***ERCC2/XPD* rs13181** | **Nephrotoxicity** | | | | | | | |
| Khrunin et al., 2010 | CTCAE grade 1-4 | Cisplatin | Adult | Ovarian cancer | 104 | 1.385 (0.526-3.644) | 0.615 | (37) |
| Khrunin et al., 2012 | CTCAE v2 grade 1-4 | Cisplatin | Adult | Ovarian cancer | 87 | N/S | ns | (38) |
| Windsor et al., 2012* | CTCAE v3 grade 3-4 | MAP | Pediatric | Osteosarcoma | 58 | 4.4 (1-18.8) | **0.044** | (3) |
| Lopes-Aguiar et al., 2017 | CTCAE v4 grade 2-5 | Cisplatin | Adult | Head and neck squamous cell carcinoma | 90 | 3.55 (1.27-9.87) | **0.01** | (39) |
| Zazuli et al., 2019 | CTCAE grade 1-4 | Cisplatin | Adult | Testicular cancer | 163 | 3.16 (1.17-8.58) | **0.02** | (40) |
| Garcia et al., 2020 | GFR decline | Cisplatin | Adult | Testicular cancer | 433 | N/S | **0.03** | (41) |
| ***ERCC2/XPD* rs1799793** | **Thrombocytopenia** | | | | | | | |
| Tibaldi et al., 2008 | CTCAE v3 grade 3-4 | Cisplatin, gemcitabine | Adult | Non-Small Cell Lung Cancer Patients | 65 | N/S | 0.35 | (42) |
| Khrunin et al., 2010 | CTCAE grade 1-4 | Cisplatin | Adult | Ovarian cancer | 87 | 4.054 (1.21-13.583) | **0.027** | (37) |
| Hattinger et al., 2016* | CTCAE v4 grade 4 | MAP | Pediatric | Osteosarcoma | 57 | 5.59 (1.66-18.87) | **0.006** | (6) |
| Lopes-Aguiar et al., 2017 | CTCAE v4 grade 1-4 | Cisplatin | Adult | Head and neck squamous cell carcinoma | 90 | N/S | ns | (39) |
| ***GGH* rs1800909[HW]** | **Hepatotoxicity** | | | | | | | |
| Hattinger et al., 2016* | CTCAE v4 grade 4 | MAP | Pediatric | Osteosarcoma | 57 | 2.87 (1.53-5.41) | **0.001** | (6) |
| ***GSTP1* rs1695** | **Leukopenia** | | | | | | | |
| Windsor et al., 2012* | CTCAE v3 grade 3-4 | Doxorubicin | Pediatric | Osteosarcoma | 58 | 7.8 (1.3-47) | **0.024** | (3) |
| Aráoz et al., 2015 | WHO grade 3-4 | MTX | Pediatric | ALL | 286 | N/S | ns | (43) |
| Hattinger et al., 2016 | CTCAE v4 grade 4 | MAP | Pediatric | Osteosarcoma | 57 | N/S | ns | (6) |

| | | | | | | | | |
|---|---|---|---|---|---|---|---|---|
| Hurkmans et al., 2020 | Leukocyte counts | MTX | Pediatric | Osteosarcoma | 113 | N/S | ns | (34) |
| ***MTHFD1* rs2236225** | **Anemia** | | | | | | | |
| Erculj et al., 2012 | CTCAE grade 2-4 | MTX | Pediatric | ALL | 167 | 0.84 (0.37-1.91) | 0.669 | (44) |
| Windsor et al., 2012* | CTCAE v3 grade 3-4 | MTX | Pediatric | Osteosarcoma | 58 | 5.4 (1-27.5) | **0.044** | (3) |
| ***MTHFR* rs1801131** | **Anemia** | | | | | | | |
| Huang et al., 2008 | Requirement of red blood cell transfusions | MTX | Pediatric | ALL | 81 | N/S | **0.043** | (45) |
| Kantar et al., 2009 | CTCAE grade 3-4 | MTX | Pediatric | ALL and NHL | 37 | N/S | **0.02** | (46) |
| Karathanasis et al., 2011 | CTCAE v4 grade 1-4 | MTX | Pediatric | ALL | 35 | N/S | 0.578 | (47) |
| Liu et al., 2011 | CTCAE v1 grade 2-4 | MTX | Pediatric | ALL | 181 | 0.52 (0.25-1.09) | 0.081 | (48) |
| Erculj et al., 2012 | CTCAE grade 2-4 | MTX | Pediatric | ALL | 167 | 0.85 (0.38-1.88) | 0.69 | (44) |
| Windsor et al., 2012* | CTCAE v3 grade 3-4 | MTX | Pediatric | Osteosarcoma | 58 | 4.6 (1.1-19.2) | **0.038** | (3) |
| Aráoz et al., 2015 | WHO grade 3-4 | MTX | Pediatric | ALL | 286 | N/S | ns | (43) |
| Yousef et al., 2019 | CTCAE v4.03 grade 3-4 | MTX | Pediatric | ALL | 64 | N/S | 0.25 | (49) |
| ***MTHFR* rs1801131** | **Leukopenia** | | | | | | | |
| Huang et al., 2008 | White blood cell count | MTX | Pediatric | ALL | 31 | N/S | 0.053 | (45) |
| van Kooten et al., 2008 | CTCAE v2 grade 3-4 | MTX | Pediatric | ALL | 88 | N/S | ns | (50) |
| Kantar et al., 2009 | CTCAE grade 3-4 | MTX | Pediatric | ALL and NHL | 37 | N/S | 0.09 | (46) |
| Faganel Kotnik et al., 2011 | Leukopenia ≥ grade 1 | MTX | Pediatric | ALL and ML | 64 | 0.14 (0.037-0.54) | **0.012** | (51) |
| Karathanasis et al., 2011 | CTCAE v4 grade 1-4 | MTX | Pediatric | ALL | 35 | N/S | 0.464 | (47) |
| Haase et al., 2012 | CTCAE grade 3-4 | MTX | Pediatric | ALL | 34 | N/S | ns | (52) |
| Suthandiram et al., 2014 | CTCAE v2 grade 1-4 | MTX | Adult | Hematological malignancies | 71 | 0.92 (0.28-23.37) | 0.9 | (53) |
| Aráoz et al., 2015 | WHO grade 3-4 | MTX | Pediatric | ALL | 286 | N/S | ns | (43) |
| Hattinger et al., 2016* | CTCAE v4 grade 4 | MAP | Pediatric | Osteosarcoma | 57 | 4.15 (1.06-16.13) | **0.04** | (6) |
| Milosevic et al., 2019 | Number of leukopenic episodes | 6-MP, MTX | Pediatric | ALL | 127 | N/S | 0.25 | (54) |
| Yousef et al., 2019 | CTCAE v4.03 grade 3-4 | MTX | Pediatric | ALL | 64 | 2.1 (0.91-5) | 0.076 | (49) |
| ***MTHFR* rs1801133** | **Nephrotoxicity** | | | | | | | |
| Kantar et al., 2009 | CTCAE grade 3-4 | MTX | Pediatric | ALL and NHL | 37 | N/S | 0.29 | (46) |
| Karathanasis et al., 2011 | CTCAE v4 grade 1-4 | MTX | Pediatric | ALL | 35 | N/S | 0.628 | (47) |
| Windsor et al., 2012* | CTCAE v3 grade 3-4 | MAP | Pediatric | Osteosarcoma | 58 | 3.1 (0.9-11.6) | 0.085 [c] | (3) |
| El Khodary et al., 2012 | Serum alpha1-microglobulin, serum creatinin, GFR | MTX | Pediatric | ALL | 40 | N/S | **<0.0001** | (55) |

| | | | | | | | | |
|---|---|---|---|---|---|---|---|---|
| Xu et al., 2018 | CTCAE grade 3-4 | MTX | Pediatric | Osteosarcoma | 109 | 1.12 (0.61-2.01) | 0.76 | (56) |
| Chae et al., 2020 | Nephrotoxicity | MTX | Pediatric | ALL | 117 | N/S | ns | (57) |
| ***NR1I2/SXR/PXR* rs3732361** | **Hepatotoxicity** | | | | | | | |
| Hegyi et al., 2017* | CTCAE v3 grade 3-4 | MTX | Pediatric | Osteosarcoma | 59 | 0.1 (0.01-0.7) | **0.014** | (33) |
| ***NR1I2/SXR/PXR* rs3732361** | **Leukopenia** | | | | | | | |
| Hegyi et al., 2017* | CTCAE v3 grade 3-4 | MTX | Pediatric | Osteosarcoma | 59 | 0.1 (0.01-0.7) | **0.013** | (33) |
| ***NR1I2/SXR/PXR* rs3814058** | **Hepatotoxicity** | | | | | | | |
| Hegyi et al., 2017* | CTCAE v3 grade 3-4 | MTX | Pediatric | Osteosarcoma | 59 | 0.3 (0.1-0.7) | **0.007** | (33) |
| ***NR1I2/SXR/PXR* rs3814058** | **Leukopenia** | | | | | | | |
| Hegyi et al., 2017* | CTCAE v3 grade 3-4 | MTX | Pediatric | Osteosarcoma | 59 | 0.3 (0.1-0.7) | **0.007** | (33) |
| ***NR1I2/SXR/PXR* rs6785049** | **Hepatotoxicity** | | | | | | | |
| Hegyi et al., 2017* | CTCAE v3 grade 3-4 | MTX | Pediatric | Osteosarcoma | 59 | 0.1 (0.01-0.7) | **0.02** | (33) |
| Hurkmans et al., 2020 | ALAT | MTX | Pediatric | Osteosarcoma | 113 | N/S | ns | (34) |
| ***NR1I2/SXR/PXR* rs6785049** | **Leukopenia** | | | | | | | |
| Hegyi et al., 2017* | CTCAE v3 grade 3-4 | MTX | Pediatric | Osteosarcoma | 59 | 0.09 (0.01-0.7) | **0.01** | (33) |
| Hurkmans et al., 2020 | Leukocyte counts | MTX | Pediatric | Osteosarcoma | 113 | N/S | ns | (34) |
| ***XPC* rs2228001** | **Infection** | | | | | | | |
| Windsor et al., 2012* | CTCAE v3 grade 3-4 | Doxorubicin | Pediatric | Osteosarcoma | 58 | 0.2 (0.06-0.8) | **0.024** | (3) |

N/S, not specified; OR, odds ratio; CTCAE, Common Terminology Criteria for Adverse Events; MAP, methotrexate, anthracycline (doxorubicin), cisplatin treatment regimen; MTX, methotrexate; ALAT, alanine aminotransferase; ALL, acute lymphatic leukemia; ML, malignant lymphoma; NHL, non-Hodgkin lymphoma

* discovery study that was identified in systematic literature search

[a] As reported by the authors

[b] Coefficient (95% CI)

[c] p=0.043 in t-test, but results from regression analysis are reported in the table for consistency

REFERENCES


(1) Caronia, D. *et al.* Effect of ABCB1 and ABCC3 polymorphisms on osteosarcoma survival after chemotherapy: a pharmacogenetic study. *PLoS One* **6**, e26091 (2011).

(2) Liu, S., Yi, Z., Ling, M., Shi, J., Qiu, Y. & Yang, S. Predictive potential of ABCB1, ABCC3, and GSTP1 gene polymorphisms on osteosarcoma survival after chemotherapy. *Tumour Biol* **35**, 9897-904 (2014).

(3) Windsor, R.E., Strauss, S.J., Kallis, C., Wood, N.E. & Whelan, J.S. Germline genetic polymorphisms may influence chemotherapy response and disease outcome in osteosarcoma: a pilot study. *Cancer* **118**, 1856-67 (2012).

(4) Yang, J., Wang, Z.G., Cai, H.Q., Li, Y.C. & Xu, Y.L. Effect of variation of ABCB1 and ABCC3 genotypes on the survival of bone tumor cases after chemotherapy. *Asian Pac J Cancer Prev* **14**, 4595-8 (2013).

(5) Li, J.Z., Tian, Z.Q., Jiang, S.N. & Feng, T. Effect of variation of ABCB1 and GSTP1 on osteosarcoma survival after chemotherapy. *Genet Mol Res* **13**, 3186-92 (2014).

(6) Hattinger, C.M. *et al.* Candidate germline polymorphisms of genes belonging to the pathways of four drugs used in osteosarcoma standard chemotherapy associated with risk, survival and toxicity in non-metastatic high-grade osteosarcoma. *Oncotarget* **7**, 61970-87 (2016).

(7) Goričar, K., Kovač, V., Jazbec, J., Zakotnik, B., Lamovec, J. & Dolžan, V. Influence of the folate pathway and transporter polymorphisms on methotrexate treatment outcome in osteosarcoma. *Pharmacogenet Genomics* **24**, 514-21 (2014).

(8) Hagleitner, M.M. *et al.* A First Step toward Personalized Medicine in Osteosarcoma: Pharmacogenetics as Predictive Marker of Outcome after Chemotherapy-Based Treatment. *Clin Cancer Res* **21**, 3436-41 (2015).

(9) Xu, L., Xia, C., Sun, Q., Sheng, F., Xiong, J. & Wang, S. Variants of FasL and ABCC5 are predictive of outcome after chemotherapy-based treatment in osteosarcoma. *J Bone Oncol* **12**, 44-8 (2018).

(10) Bhuvaneshwar, K. *et al.* Genome sequencing analysis of blood cells identifies germline haplotypes strongly associated with drug resistance in osteosarcoma patients. *BMC Cancer* **19**, 357 (2019).

(11) Koster, R. *et al.* Genome-wide association study identifies the GLDC/IL33 locus associated with survival of osteosarcoma patients. *Int J Cancer* **142**, 1594-601 (2018).

(12) Lin, Q., Han, J., Sun, Q., Wen, L. & Wang, S. Functional variant of IL33 is associated with survival of osteosarcoma patients. *J Bone Oncol* **20**, 100270 (2020).

(13) Yang, L.M., Li, X.H. & Bao, C.F. Glutathione S-transferase P1 and DNA polymorphisms influence response to chemotherapy and prognosis of bone tumors. *Asian Pac J Cancer Prev* **13**, 5883-6 (2012).

(14) Zhang, S.L., Mao, N.F., Sun, J.Y., Shi, Z.C., Wang, B. & Sun, Y.J. Predictive potential of glutathione S-transferase polymorphisms for prognosis of osteosarcoma patients on chemotherapy. *Asian Pac J Cancer Prev* **13**, 2705-9 (2012).

(15) Teng, J.W., Yang, Z.M., Li, J. & Xu, B. Predictive role of Glutathione S-transferases (GSTs) on the prognosis of osteosarcoma patients treated with chemotherapy. *Pak J Med Sci* **29**, 1182-6 (2013).

(16) Jabeen, S., Holmboe, L., Alnaes, G.I., Andersen, A.M., Hall, K.S. & Kristensen, V.N. Impact of genetic variants of RFC1, DHFR and MTHFR in osteosarcoma patients treated with high-dose methotrexate. *Pharmacogenomics J* **15**, 385-90 (2015).

(17) Hildebrandt, M.A.T. *et al.* Hypertension Susceptibility Loci are Associated with Anthracycline-related Cardiotoxicity in Long-term Childhood Cancer Survivors. *Sci Rep* **7**, 9698 (2017).

(18) Wang, X. *et al.* CELF4 Variant and Anthracycline-Related Cardiomyopathy: A Children's Oncology Group Genome-Wide Association Study. *J Clin Oncol* **34**, 863-70 (2016).

(19) Leger, K.J. *et al.* Clinical and Genetic Determinants of Cardiomyopathy Risk among Hematopoietic Cell Transplantation Survivors. *Biol Blood Marrow Transplant* **22**, 1094-101 (2016).
(20) Ruiz-Pinto, S. *et al.* Exome array analysis identifies GPR35 as a novel susceptibility gene for anthracycline-induced cardiotoxicity in childhood cancer. *Pharmacogenet Genomics* **27**, 445-53 (2017).
(21) Wang, X. *et al.* Hyaluronan synthase 3 variant and anthracycline-related cardiomyopathy: a report from the children's oncology group. *J Clin Oncol* **32**, 647-53 (2014).
(22) Sági, J.C. *et al.* Possible roles of genetic variations in chemotherapy related cardiotoxicity in pediatric acute lymphoblastic leukemia and osteosarcoma. *BMC Cancer* **18**, 704 (2018).
(23) Aminkeng, F. *et al.* A coding variant in RARG confers susceptibility to anthracycline-induced cardiotoxicity in childhood cancer. *Nat Genet* **47**, 1079-84 (2015).
(24) Serie, D.J. *et al.* Genome-wide association study of cardiotoxicity in the NCCTG N9831 (Alliance) adjuvant trastuzumab trial. *Pharmacogenet Genomics* **27**, 378-85 (2017).
(25) Schneider, B.P. *et al.* Genome-Wide Association Study for Anthracycline-Induced Congestive Heart Failure. *Clin Cancer Res* **23**, 43-51 (2017).
(26) Park, B., Sim, S.H., Lee, K.S., Kim, H.J. & Park, I.H. Genome-wide association study of genetic variants related to anthracycline-induced cardiotoxicity in early breast cancer. *Cancer Sci* **111**, 2579-87 (2020).
(27) Visscher, H. *et al.* Genetic variants in SLC22A17 and SLC22A7 are associated with anthracycline-induced cardiotoxicity in children. *Pharmacogenomics* **16**, 1065-76 (2015).
(28) Visscher, H. *et al.* Pharmacogenomic prediction of anthracycline-induced cardiotoxicity in children. *J Clin Oncol* **30**, 1422-8 (2012).
(29) Visscher, H. *et al.* Validation of variants in SLC28A3 and UGT1A6 as genetic markers predictive of anthracycline-induced cardiotoxicity in children. *Pediatr Blood Cancer* **60**, 1375-81 (2013).
(30) Vulsteke, C. *et al.* Clinical and genetic risk factors for epirubicin-induced cardiac toxicity in early breast cancer patients. *Breast Cancer Res Treat* **152**, 67-76 (2015).
(31) Reichwagen, A. *et al.* Association of NADPH oxidase polymorphisms with anthracycline-induced cardiotoxicity in the RICOVER-60 trial of patients with aggressive CD20(+) B-cell lymphoma. *Pharmacogenomics* **16**, 361-72 (2015).
(32) Hertz, D.L. *et al.* Evidence for association of SNPs in ABCB1 and CBR3, but not RAC2, NCF4, SLC28A3 or TOP2B, with chronic cardiotoxicity in a cohort of breast cancer patients treated with anthracyclines. *Pharmacogenomics* **17**, 231-40 (2016).
(33) Hegyi, M. *et al.* Pharmacogenetic analysis of high-dose methotrexate treatment in children with osteosarcoma. *Oncotarget* **8**, 9388-98 (2017).
(34) Hurkmans, E.G.E. *et al.* Analysis of Drug Metabolizing Gene Panel in Osteosarcoma Patients Identifies Association Between Variants in SULT1E1, CYP2B6 and CYP4F8 and Methotrexate Levels and Toxicities. *Front Pharmacol* **11**, 1241 (2020).
(35) Sharifi, M.J., Bahoush, G., Zaker, F., Ansari, S., Rafsanjani, K.A. & Sharafi, H. Association of -24CT, 1249GA, and 3972CT ABCC2 gene polymorphisms with methotrexate serum levels and toxic side effects in children with acute lymphoblastic leukemia. *Pediatr Hematol Oncol* **31**, 169-77 (2014).
(36) Gervasini, G., de Murillo, S.G., Jimenez, M., de la Maya, M.D. & Vagace, J.M. Effect of polymorphisms in transporter genes on dosing, efficacy and toxicity of maintenance therapy in children with acute lymphoblastic leukemia. *Gene* **628**, 72-7 (2017).
(37) Khrunin, A.V., Moisseev, A., Gorbunova, V. & Limborska, S. Genetic polymorphisms and the efficacy and toxicity of cisplatin-based chemotherapy in ovarian cancer patients. *Pharmacogenomics J* **10**, 54-61 (2010).
(38) Khrunin, A. *et al.* Pharmacogenomics of cisplatin-based chemotherapy in ovarian cancer patients of different ethnic origins. *Pharmacogenomics* **13**, 171-8 (2012).

(39) Lopes-Aguiar, L. *et al.* XPD c.934G>A polymorphism of nucleotide excision repair pathway in outcome of head and neck squamous cell carcinoma patients treated with cisplatin chemoradiation. *Oncotarget* **8**, 16190-201 (2017).
(40) Zazuli, Z. *et al.* Outcome Definition Influences the Relationship Between Genetic Polymorphisms of ERCC1, ERCC2, SLC22A2 and Cisplatin Nephrotoxicity in Adult Testicular Cancer Patients. *Genes (Basel)* **10**, (2019).
(41) Garcia, S.L. *et al.* Prediction of Nephrotoxicity Associated With Cisplatin-Based Chemotherapy in Testicular Cancer Patients. *JNCI Cancer Spectr* **4**, pkaa032 (2020).
(42) Tibaldi, C. *et al.* Correlation of CDA, ERCC1, and XPD polymorphisms with response and survival in gemcitabine/cisplatin-treated advanced non-small cell lung cancer patients. *Clin Cancer Res* **14**, 1797-803 (2008).
(43) Araoz, H.V. *et al.* Pharmacogenetic studies in children with acute lymphoblastic leukemia in Argentina. *Leuk Lymphoma* **56**, 1370-8 (2015).
(44) Erculj, N., Kotnik, B.F., Debeljak, M., Jazbec, J. & Dolzan, V. Influence of folate pathway polymorphisms on high-dose methotrexate-related toxicity and survival in childhood acute lymphoblastic leukemia. *Leuk Lymphoma* **53**, 1096-104 (2012).
(45) Huang, L., Tissing, W.J., de Jonge, R., van Zelst, B.D. & Pieters, R. Polymorphisms in folate-related genes: association with side effects of high-dose methotrexate in childhood acute lymphoblastic leukemia. *Leukemia* **22**, 1798-800 (2008).
(46) Kantar, M. *et al.* Methylenetetrahydrofolate reductase C677T and A1298C gene polymorphisms and therapy-related toxicity in children treated for acute lymphoblastic leukemia and non-Hodgkin lymphoma. *Leuk Lymphoma* **50**, 912-7 (2009).
(47) Karathanasis, N.V., Stiakaki, E., Goulielmos, G.N. & Kalmanti, M. The role of the methylenetetrahydrofolate reductase 677 and 1298 polymorphisms in Cretan children with acute lymphoblastic leukemia. *Genet Test Mol Biomarkers* **15**, 5-10 (2011).
(48) Liu, S.G., Li, Z.G., Cui, L., Gao, C., Li, W.J. & Zhao, X.X. Effects of methylenetetrahydrofolate reductase gene polymorphisms on toxicities during consolidation therapy in pediatric acute lymphoblastic leukemia in a Chinese population. *Leuk Lymphoma* **52**, 1030-40 (2011).
(49) Yousef, A.M., Farhad, R., Alshamaseen, D., Alsheikh, A., Zawiah, M. & Kadi, T. Folate pathway genetic polymorphisms modulate methotrexate-induced toxicity in childhood acute lymphoblastic leukemia. *Cancer Chemother Pharmacol* **83**, 755-62 (2019).
(50) van Kooten Niekerk, P.B., Schmiegelow, K. & Schroeder, H. Influence of methylene tetrahydrofolate reductase polymorphisms and coadministration of antimetabolites on toxicity after high dose methotrexate. *Eur J Haematol* **81**, 391-8 (2008).
(51) Faganel Kotnik, B., Grabnar, I., Bohanec Grabar, P., Dolzan, V. & Jazbec, J. Association of genetic polymorphism in the folate metabolic pathway with methotrexate pharmacokinetics and toxicity in childhood acute lymphoblastic leukaemia and malignant lymphoma. *Eur J Clin Pharmacol* **67**, 993-1006 (2011).
(52) Haase, R. *et al.* High dose methotrexate treatment in childhood ALL: pilot study on the impact of the MTHFR 677C>T and 1298A>C polymorphisms on MTX-related toxicity. *Klin Padiatr* **224**, 156-9 (2012).
(53) Suthandiram, S. *et al.* Effect of polymorphisms within methotrexate pathway genes on methotrexate toxicity and plasma levels in adults with hematological malignancies. *Pharmacogenomics* **15**, 1479-94 (2014).
(54) Milosevic, G. *et al.* Influence of variants in folate metabolism genes on 6-mercaptopurine induced toxicity during treatment for childhood acute lymphocytic leukemia. *J BUON* **24**, 2075-83 (2019).
(55) El-Khodary, N.M., El-Haggar, S.M., Eid, M.A. & Ebeid, E.N. Study of the pharmacokinetic and pharmacogenetic contribution to the toxicity of high-dose methotrexate in children with acute lymphoblastic leukemia. *Med Oncol* **29**, 2053-62 (2012).

(56) Xu, L., Wang, L., Xue, B. & Wang, S. MTHFR variant is associated with high-dose methotrexate-induced toxicity in the Chinese osteosarcoma patients. *J Bone Oncol* **13**, 143-7 (2018).

(57) Chae, H. *et al.* Influence of plasma methotrexate level and MTHFR genotype in Korean paediatric patients with acute lymphoblastic leukaemia. *J Chemother* **32**, 251-9 (2020).
